# Palladium-catalyzed synthesis of N-arylated carbazoles using anilines and cyclic diaryliodonium salts

**DOI:** 10.3762/bjoc.9.136

**Published:** 2013-06-21

**Authors:** Stefan Riedmüller, Boris J Nachtsheim

**Affiliations:** 1 Merck KGaA, Performance Materials Division, Location F61/391, Frankfurter Straße 250, 64293 Darmstadt, Germany; 2 Institut für Organische Chemie, Eberhard Karls Universität Tübingen, Auf der Morgenstelle 18, 72076 Tübingen, Germany

**Keywords:** amination, carbazoles, hypervalent, iodine, iodonium salts

## Abstract

The direct synthesis of N-arylated carbazoles through a palladium-catalyzed amination of cyclic iodonium salts with anilines is described. In particular, electron-poor aniline derivatives reacted smoothly with only 5 mol % of Pd(OAc)_2_ as catalyst to give the desired products in up to 71% yield. Furthermore, the reactivity of cyclic iodonium salts is compared with the reactivity of the corresponding cyclic bromonium analogues.

## Introduction

**Figure 1:**
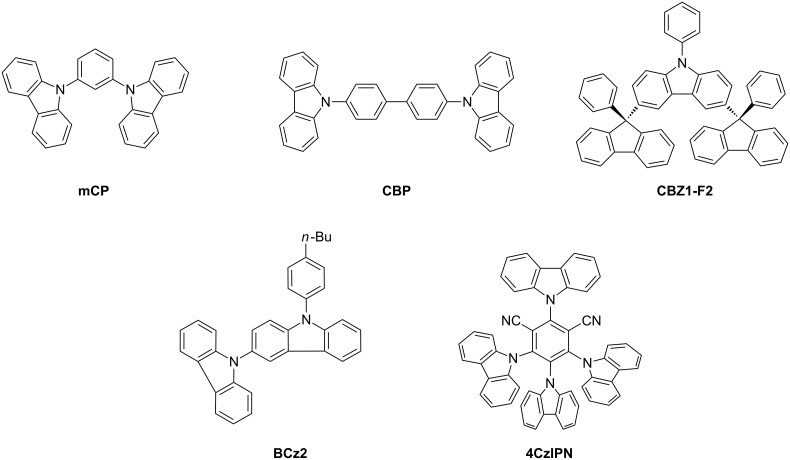
Representative examples of carbazoles with hole-transport, host or luminescent properties.

Carbazoles play an important role as core structural elements in natural products (e.g., alkaloids) and pharmaceuticals [1]. In addition, the carbazole motif constitutes an immense class of materials in the rapidly growing field of molecular electronics. In particular *N*-arylcarbazoles have promising electroluminescent properties and have subsequently found diverse applications as hole-transport, or as host or luminescent-materials in electronic devices (OLEDs) (Figure 1) [2–7]. Representative examples are the host molecules **mCP**, **CBP** and **CBZ1-F2**, the hole transporter **BCz2** [8] or the recently described thermally activated delayed fluorescence (TADF) emitter **4CzIPN** [9].

**Scheme 1:**
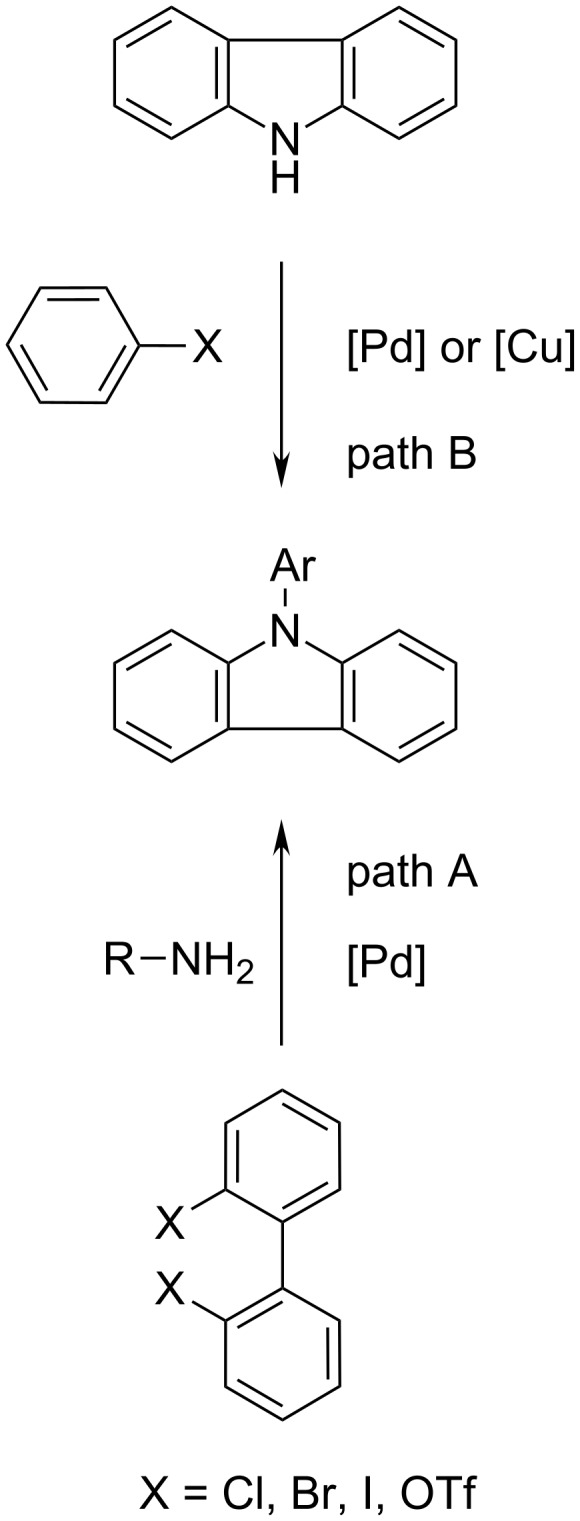
Synthetic access to N-arylated carbazoles.

Therefore, the efficient synthesis of N-arylated carbazoles is an attractive goal and numerous synthetic methods are known so far from the literature. The main synthetic routes are shown in Scheme 1. The majority are transition-metal mediated. Starting from functionalized 2,2**'**-biphenyls (path A) [10–13] or the direct arylation [14–15] of the free NH-functionality of carbazole (path B).

In the past decade, hypervalent iodine chemistry has undergone a renaissance and has developed to become a powerful area in synthetic organic chemistry. Open-chained iodonium salts are well explored in transition-metal-mediated reactions to construct new C–N bonds [16–19], whereas examples dealing with cyclic iodonium salts are underrepresented [20]. Our group is interested in the development of new C–X coupling strategies via (hypo)iodite or hypervalent iodine catalysis [21–23]. Here, we wish to present an alternative Pd-catalyzed method for the construction of N-substituted carbazoles based on a stable, cyclic iodonium salt and electron-deficient anilines [24–25].

**Scheme 2:**
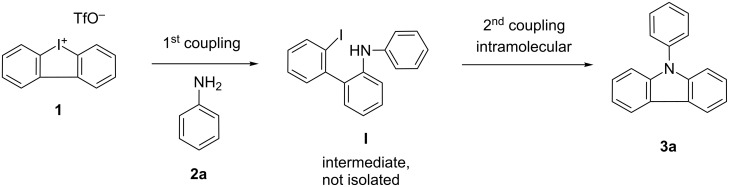
Proposed mechanistic motivation towards the formation of **3a**.

In the initial C–N bond-forming step of this cascade reaction, a ring opening of the cyclic iodonium salt through the amine is proposed to give 2'-iodobiphenyl-2-phenylamine (**I**). In a second, Pd-mediated intramolecular cross-coupling, 9-phenyl-9*H*-carbazole (**3a**) should be observed (Scheme 2).

## Results and Discussion

First, we decided to prepare cyclic iodonium salt **1** as the triflate salt, to avoid unwanted side-reactions in solution with a concurrent nucleophilic counterion [26]. However, **1** was synthesized from 2-iodobiphenyl according to the established one-pot procedure for the synthesis of diaryliodonium triflates [27–28] by Olofsson and co-workers (Supporting Information File 1).

After promising initial experiments, we systematically optimized the reaction conditions of a reaction between aniline and cyclic iodonium salt **1** (Table 1). Various reaction parameters, in particular the Pd catalyst, catalyst loading, the phosphine ligand, and the temperature had a significant influence on the outcome of this transformation. Starting with Pd_2_(dba)_3_, SPhos (2 mol % and 4 mol %, respectively), NaO*t-*Bu or Cs_2_CO_3_ in toluene resulted only in trace amounts of **3a** (Table 1, entry 1 and 2). After increasing the catalyst/ligand ratio (palladium to phosphine 5 mol % and 10 mol %, respectively) and using Cs_2_CO_3_ as the base, **3a** could be isolated in 35% yield (Table 1, entry 3). Next, we varied the phosphine ligands (Table 1, entries 4–9). Xantphos was the most efficient bidentate ligand yielding **3a** in 46% yield (Table 1, entry 6). Xylenes as the solvent, for example *p*-xylene, were also suitable for the reaction; in contrast to DME, where the yield slightly decreases (Table 1, entry 11). We also tested *t*-*Bu*-Xantphos (Table 1, entry 8) as a common ligand with a higher bite angle. However, this Xantphos derivative is totally inefficient in our coupling reaction. Dppf and BINAP gave very similar results in isolated yield (natural bite angle of the phosphines 99° and 93°, respectively) (Table 1, entry 5 and 7). When using bidentate ligands with bite angles higher than 100° (DPEphos 104°, Xantphos 108°) the reaction is more efficient and the yield increases significantly. Next, we asked ourselves, whether other palladium salts could be equal or better in efficiency and yield. Changing Pd_2_(dba)_3_ to Pd(OAc)_2_ had no significant impact on product yields (Table 1, entry 12). Further increase of the catalyst ratio from 5 to 10 mol % had little effect (Table 1, entry 15).

**Table 1:** Optimizing the reaction conditions^a^.

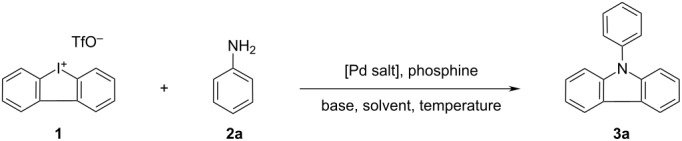							

entry	catalyst 5 mol %	phosphine 10 mol %	base	solvent	time [h]	temp [°C]	yield [%]^b^

1^c^	Pd_2_dba_3_	SPhos	NaO*t*-Bu	toluene	19	105	trace^d^
2^c^	Pd_2_dba_3_	SPhos	Cs_2_CO_3_	toluene	19	105	trace^d^
3	Pd_2_dba_3_	SPhos	Cs_2_CO_3_	toluene	5	105	35
4	Pd_2_dba_3_	P(*t*-Bu)_3_	Cs_2_CO_3_	toluene	4	105	34
5	Pd_2_dba_3_	dppf	Cs_2_CO_3_	toluene	13	105	14
6	Pd_2_dba_3_	Xantphos	Cs_2_CO_3_	toluene	14	105	46
7	Pd_2_dba_3_	BINAP	Cs_2_CO_3_	*p*-xylene	16	125	16
8	Pd_2_dba_3_	*t*-Bu-Xantphos	Cs_2_CO_3_	*p*-xylene	16	125	trace^d^
9	Pd_2_dba_3_	DPE-Phos	Cs_2_CO_3_	*p*-xylene	14	125	34
10	Pd_2_dba_3_	Xantphos	Cs_2_CO_3_	*p*-xylene	12	125	42
11	Pd_2_dba_3_	Xantphos	Cs_2_CO_3_	DME	13	79	39
12	Pd(OAc)_2_	Xantphos	Cs_2_CO_3_	*p*-xylene	2.5	125	45^e^
13	Pd(OAc)_2_	Xantphos	Cs_2_CO_3_	DME	3	79	12
14	Pd(OAc)_2_	Xantphos	NaO*t*-Bu	*p*-xylene	3	125	trace^d^
15^f^	Pd(OAc)_2_	Xantphos	Cs_2_CO_3_	*p*-xylene	4	126	51

^a^All reactions were run using iodonium salt **1** (0.35 mmol), 1.2 equiv of aniline **2a**, 2.7 equiv of base, and 5 mL of solvent. ^b^Isolated yield after column chromatography. ^c^2 mol % Pd_2_dba_3_ and 4 mol % SPhos were used. ^d^Product not isolated. ^e^1.0 equiv of aniline **2a** was used. ^f^10 mol % Pd(OAc)_2_ and 20 mol % Xantphos were used. Pd_2_dba_3_ = tris(dibenzylideneacetone)dipalladium(0), SPhos = 2-dicyclohexylphosphino-2',6'-dimethoxybiphenyl, dppf = 1,1'-bis(diphenylphosphino)ferrocene, Xantphos = 9,9-dimethyl-4,5-bis(diphenylphosphino)xanthene, BINAP = 2,2'-bis(diphenylphosphino)-1,1'-binaphthyl, *t*-Bu-Xantphos = 4,5-bis(di-*tert*-butylphosphino)-9,9-dimethylxanthene, DPEphos = bis[(2-diphenylphosphino)phenyl] ether.

**Figure 2:**
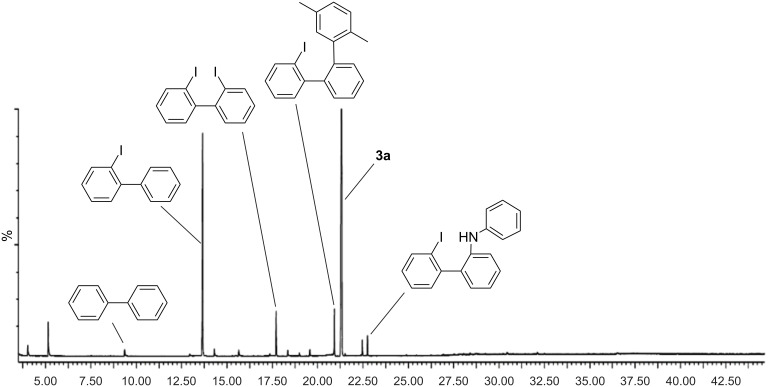
FID chromatogram of the reaction mixture. Only the most intense peaks were structurally assigned. x-Axis = retention time.

Next, we decided to analyze the byproducts of this reaction by GC–MS analysis. For this experiment the reaction was performed according to conditions given in entry 12, Table 1. Besides the desired product **3a** (56%), we could identify the masses of 2-iodobiphenyl (22%), 2,2'-diiodobiphenyl (4%), 2-(2,5-dimethylphenyl)-2'-iodobiphenyl (5%), and 2'-iodo-*N*-phenylbiphenyl-2-amine (<2%) in significant amounts (Figure 2).

After a deeper literature research we came to the conclusion, that those byproducts should probably not only arise from side reactions within the catalytic cycle (for instance, 2-iodobiphenyl from β-H elimination) but also from homolytic or heterolytic decomposition pathways of the diaryliodonium salt (2-iodobiphenyl, 2,2'-diiodobiphenyl, and 2-(2,5-dimethylphenyl)-2'-iodobiphenyl) [29–31]. To further verify these observations, we reacted **1** in the presence of aniline (**2a**) for three days at elevated temperature without adding a Pd catalyst. Again, after GC–MS analysis, we could detect 2-iodobiphenyl, 2,2'-diiodobiphenyl, 2-(2,5-dimethylphenyl)-2'-iodobiphenyl, and 2'-iodo-*N*-phenylbiphenyl-2-amine in the absence of any produced **3a**. Contrary to that observation, when we conducted an analogous experiment without aniline, we observed no decomposition or byproduct formation. These results led us to conclude that byproduct formation is, at least partially, induced through the nucleophilic and/or basic nature of aniline [26,32–35]. We therefore had to accept that a significant amount of byproducts are formed during the formation of **3a**, reducing our isolated yield.

**Figure 3:**
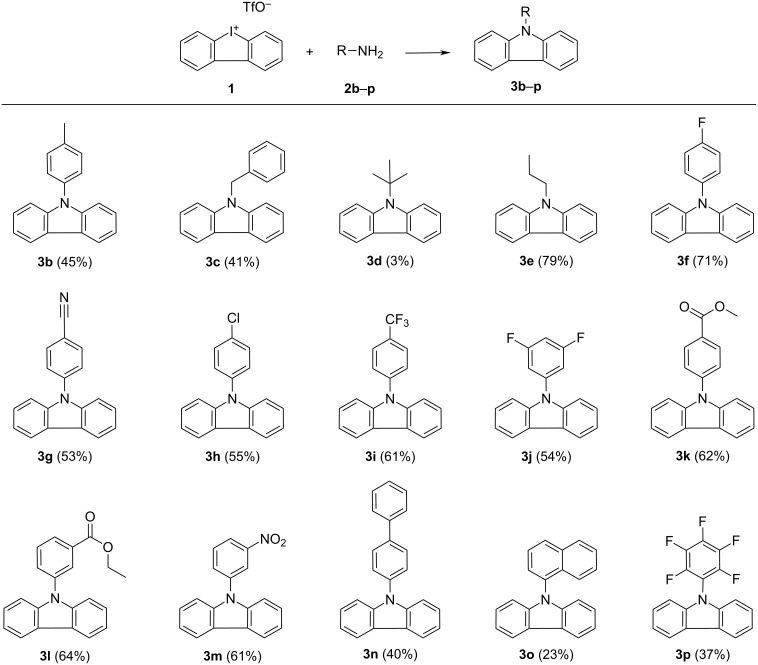
Substrate scope. All reactions were performed using iodonium salt **1** (0.35 mmol), 1.2 equiv of primary amine **2**, 2.7 equiv of Cs_2_CO_3_, 5 mol % Pd(OAc)_2_, 10 mol % Xantphos, and 5–8 mL *p*-xylene at 125 °C. Reaction times 2–4 h. Isolated yields are given in parentheses.

After we had gained a better understanding about byproduct formation, we decided to explore various substituted anilines under our optimized reaction conditions (Figure 3). Electron-rich *p*-toluidine (**2b**) gave the corresponding carbazole **3b** in only 45% yield. Comparable results were obtained when benzylamine (**2c**) was used (41% yield). Even more electron-donating aniline derivatives, such as *p*-anisidine (*p-*methoxyaniline), resulted in the formation of trace amounts of the carbazole product (not shown). The aliphatic primary amines *tert*-butylamine (**2d**) and propylamine (**2e**) were also investigated. Amine **2d** was completely inefficient and yielded 3% of **3d** compared to a 79% yield of **3e**, when using propylamine. However, when electron-withdrawing anilines were utilized, isolated yields of the corresponding carbazole increased significantly. As examples, *p*-cyano-, *p*-chloro- or *p*-COOMe-substituted anilines yielded **3g**, **3h**, and **3k** in 53%, 55% and 62% yield; *p*-fluoroaniline yielded **3f** in 71%. Other fluorine-substituted anilines, such as 4-(trifluoromethyl)aniline (**2i**) or 3,5-difluoroaniline (**2j**), were also suitable with slightly decreased yields of **3i** (61%) and **3j** (54%), respectively.

Furthermore, meta-substituted anilines **2l** and **2m** were tested, giving the N-arylated carbazoles **3l** and **3m** in good yields of 64% and 61%, respectively. In general, the use of fluorine-substituted anilines showed the best results so far in this study. However, with the perfluorinated derivative **2p**, the isolated yield of **3p** was diminished to 37%. Furthermore, we used our protocol to synthesize the *N*-arylcarbazole based electronic materials **3n** and **3o** in 40% (**3n**) and 23% (**3o**) yield.

**Scheme 3:**
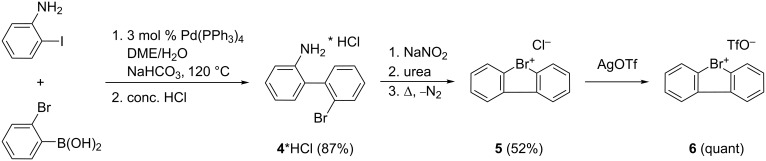
Synthesis of dibenzo[*b*,*d*]bromolium trifluoromethanesulfonate (**6**).

After an extensive exploration of the reaction conditions and the substrate scope with iodonium salt **1**, we wanted to compare our results with the corresponding bromonium analogue. Cyclic diarylbromonium salts are considerably less explored than their iodonium congeners as can be seen by only a handful of synthetic methods described in the literature [32,36–41]. In general, bromonium salts are more reactive but have similar reaction behaviour [32,36]. Thus they could be helpful substrates for the synthesis of *N*-arylcarbazoles from anilines. With this in mind, we initially focussed on the synthesis of dibenzo[*b*,*d*]bromolium chloride (**5**) using a procedure published by Sandin and Hay in 1952 [41] (Scheme 3).

**Scheme 4:**
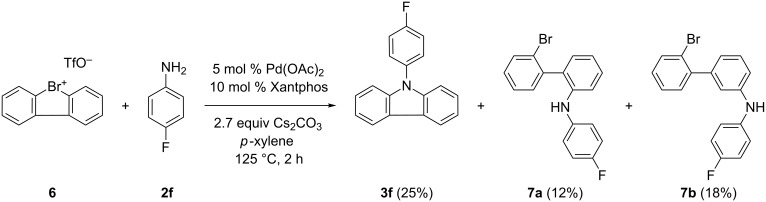
Dibenzo[*b*,*d*]bromolium trifluoromethanesulfonate (**6**) and *p*-fluoroaniline (**2f**) to construct carbazole **3f**.

The biphenyl derivative 4*HCl was prepared by Suzuki coupling of 2-iodoaniline and 2-bromophenylboronic acid. Diazotation of 4*HCl and cyclization gave the cyclic diarylbromonium chloride **5** as an off-white powder in good isolated yield (52%) (Supporting Information File 1) [41]. Finally, **5** could be converted into the corresponding diarylbromonium triflate **6** with silver trifluoromethanesulfonate in quantitative yield (Supporting Information File 1). To verify whether dibenzo[*b*,*d*]bromolium trifluoromethanesulfonate (**6**) is indeed a more reactive surrogate for the construction of N-arylated carbazoles, we reacted **6** with *p*-fluoroaniline (**2f**) according to our previously described optimized conditions (Scheme 4).

However, **3f** was obtained in only 25% yield (Scheme 4), compared to 71% when using the corresponding diaryliodonium salt **1** (Figure 3). Apart from the desired product, we were able to isolate two byproducts from the crude reaction mixture. After a systematic structure determination by one- and two-dimensional NMR techniques as well as mass spectrometry, we elucidated the two byproducts as the two regioisomers 2'-bromo-*N*-(4-fluorophenyl)biphenyl-2-amine (**7a**, 12%) and 2'-bromo-*N*-(4-fluorophenyl)biphenyl-3-amine (**7b**, 18%) (Scheme 4). Compound **7a** was either formed during the catalytic cycle as a reaction intermediate, which had not reacted further to the final product **3f**, or is the result of a nucleophilic attack, caused by the nucleophilic nature of aniline **2f** at the electrophilic ipso-position in **6**. The formation of the other regioisomer **7b**, is not evident at first glance. One plausible explanation could be the emergence of a benzyne intermediate during synthesis, generated by β-elimination using aniline as a base. Subsequent nucleophilic trapping of the benzyne with aniline, this time reacting as a nucleophile, results in the formation of **7b**. A very similar reactivity was described recently for a nitro-substituted diarylbromonium salt [42]. However, the results of these experiments demonstrate, that the higher reactivity of diarylbromonium salts towards nucleophilic ring opening is accompanied, to a significant degree, by an undesired β-elimination pathway, leading to more complex reaction mixtures and subsequently lower yields of the desired *N-*arylcarbazole.

## Conclusion

In summary, we have developed a novel synthesis of synthetically highly useful *N*-arylcarbazoles starting from cyclic diaryliodonium salts by a ring opening/Buchwald-amination cascade using anilines and aliphatic amines as nitrogen-containing substrates. With 5 mol % of Pd(OAc)_2_ the desired *N*-arylcarbazoles could be isolated in up to 71% yield. Finally, the corresponding cyclic diarylbromonium derivatives were tested in the same reaction. Significantly lower yields were observed due to undesired side reactions involving benzyne intermediates by β-elimination.

## Supporting Information

File 1 Experimental procedures and data of characterization of the described compounds.
