# Supplementary material for: Palladium-catalyzed synthesis of N-arylated carbazoles using anilines and cyclic diaryliodonium salts
Source: Beilstein J Org Chem. 2013 Jun 21;9:1202–9. doi: 10.3762/bjoc.9.136 (PMC3701385; doi:10.3762/bjoc.9.136)

Supporting Information

for

Palladium-catalyzed synthesis of N-arylated carbazoles using anilines and cyclic diaryliodonium salts

Stefan Riedmüller[1,2] and Boris J. Nachtsheim*[2]

Address: [1]Merck KGaA, Performance Materials Division, Location F61/391, Frankfurter Straße 250, 64293 Darmstadt, Germany and [2]Institut für Organische Chemie, Eberhard Karls Universität Tübingen, Auf der Morgenstelle 18, 72076 Tübingen, Germany

Email: Boris J. Nachtsheim - boris.nachtsheim@uni-tuebingen.de

* Corresponding author

Experimental procedures and data of characterization of the described compounds

Contents:

I. General methods, solvents and reagents S2

II. General experimental procedures S3

III. Characterization data S6

IV. References S14

V. Copies of ^{1}H NMR, ^{13}C NMR and ^{19}F NMR spectra S15

I. General methods

^{1}H NMR spectra were recorded on an Avance 400 MHz and 500 MHz instrument in deuterated chloroform $CDCl_3$ or DMSO-d_6. Chemical shifts (δ) are given in parts per million (ppm). ^{1}H NMR spectra were referenced to the residual hydrogen signal in $CDCl_3$ at δ = 7.26 ppm or to the residual hydrogen signal at δ = 2.50 ppm when measured in DMSO-d_6. ^{13}C NMR spectra were recorded on 100 MHz and 126 MHz and were fully proton and ^{19}F decoupled (if necessary) by broad-band decoupling. ^{13}C NMR spectra were referenced to the $CDCl_3$ triplet signal at δ = 77.16 ppm or to the septet signal at δ = 39.52 ppm in DMSO-d_6. ^{19}F NMR spectra were recorded at 376.46 MHz with no decoupling on an Avance 400 MHz instrument. The following abbreviations were used to describe splitting patterns: br = broad, s = singlet, d = doublet, t = triplet, q = quartet, ddd = doublet of doublet of doublet, dt = doublet of triplet, td = triplet of doublet, ttt = triplet of triplet of triplet, sext = sextet, m = multiplet. Coupling constants *J* are given in Hz. Mass spectra were recorded on a Varian Saturn 2000 with a Varian GC 3800 unit (column: CP Sil 8 CB 32 m x 0.25 mm) or on a Waters GCT Premier instrument (column: VF-5ms 30 m x 0.25 mm ID DF = 0.25 μm) by using EI method. HRMS were measured on a Thermo Fisher Scientific LTQ-Orbitrap-XL. IR spectra were recorded on JASCO FT/IR-4100. A selection of signals is given in reciprocal centimeters (cm^{-1}). Elemental analysis was performed on a Hekatech GmbH CHNS analysator. Thin-layer chromatography was performed on fluorescence-indicator-marked precoated silica gel 60 plates (Merck KGaA, TLC Silica gel 60 F_{254}). Column chromatography was performed on silica gel (0.063–0.200 mm). All reactions were carried out under argon atmosphere. Reagents and solvents were added by syringe/septum techniques.

Solvents and reagents

For all conversions solvents were used as received from their suppliers (Merck KGaA, Merck Schuchardt OHG, Alfa Aesar) and were degassed before use. Solvents for column chromatography (heptane, dichloromethane) were used as received from suppliers. 2-iodobiphenyl [1] and 2'-bromo-[1,1'-biphenyl]-2-amine [2] were prepared according to the literature procedure. Dibenzo[*b*,*d*]bromolium chloride (**5**) [3] was synthesized by the literature procedure from **4***HCl and sodium nitrate.

Unless otherwise stated, all other commercially available substances and reagents were used as received from their suppliers (ABCR, Merck KGaA, Merck Schuchardt OHG, HEREAUS, Sigma Aldrich) without further purification.

II. General experimental procedures

Dibenzo[*b,d*]iodol-5-ium trifluoromethanesulfonate (1)

Compound **1** was synthesized according to Olofssons one-pot synthesis of diaryliodonium triflates [4,5] with a slightly modified molar ratio. 3-Chloroperoxybenzoic acid (2.80 g, 0.013 mol, 1.25 equiv) was dissolved in 40 mL dichloromethane at room temperature. To this solution 2-iodobiphenyl (1.69 mL, 0.01 mol, 1.0 equiv) was added. The reaction mixture was cooled down to 0 °C and trifluoromethanesulfonic acid (1.79 mL, 0.02 mol, 2.00 equiv) was slowly added. The stirred solution was allowed to warm to room temperature overnight. All volatiles were then removed in vacuo. The remaining crude residue was treated with cold diethyl ether and was subsequently filtered, washed several times with additional cold diethyl ether and dried in vacuo. 3.96 g (92%) dibenzo[*b,d*]iodol-5-ium trifluoromethanesulfonate (**1**) was isolated as an off-white powder.

Optimization studies towards the formation of **1**:

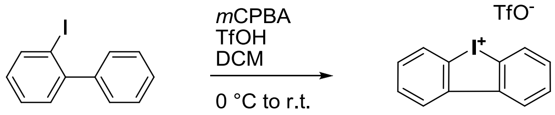

1

entry	*m*CPBA (equiv)	TfOH (equiv)	yield (%)
1	2.00	3.0	**94**
2	1.50	3.0	**94**
3	1.25	2.0	**92**

Dibenzo[*b,d*]bromolium chloride (5)

To 2'-bromo-[1,1'-biphenyl]-2-amine (4.44 g, 0.018 mol), concentrated hydrochloric acid (1.5 mL, 0.018 mol, 1.0 equiv) was added and stirred overnight. In a 500 mL three-necked round-bottomed flask, the hydrochloride of 2'-bromo-[1,1'-biphenyl]-2-amine (**4***HCl) was dissolved in boiling 10% hydrochloric acid (200 mL). The obtained solution was cooled down to ice-bath temperature (0–5 °C) and treated with sodium nitrite (2.5 g, 0.036 mol, 2.0 equiv) in 50 mL water. The dark orange solution was stirred for an additional hour at low temperature, after which urea (2.5 g, 0.042 mol, 2.3 equiv) was added at the same temperature and stirring continued for an additional hour. The solution was heated up to elevated temperature until gas evolution ceased. Activated charcoal was added to the hot reaction mixture and filtered hot. The resulting filtrate was cooled in an ice-bath giving a voluminous precipitate, which was filtered, suspended, and washed with water several times. The crude product was finally suspended in cold *tert*-butyl methyl ether to remove trace amounts of unreacted starting material. The product was dried in vacuo to give the target compound (2.505 g, 52%) as an off-white powder.

Dibenzo[*b,d*]bromolium trifluoromethanesulfonate (6)

Dibenzo[*b,d*]bromolium chloride (**5**) (0.7 g, 0.0026 mol) was dissolved in 70 mL MeOH at room temperature. To this solution, silver trifluoromethanesulfonate (0.739 g, 0.003 mol) was added, immediately forming a colorless precipitate. The suspension was then stirred for half an hour at ambient temperature. The suspension was filtered, the separated precipitate washed with MeOH, and the filtrate evaporated under reduced pressure giving 1.016 g (quant) of **6** as fine beige needles.

General procedure for the double amination of dibenzo[*b,d*]iodol-5-ium trifluoromethanesulfonate (1)

Dibenzo[*b*,*d*]iodol-5-ium trifluoromethanesulfonate (**1**, 0.150 g, 0.35 mmol, 1.0 equiv), cesium carbonate (0.311 g, 0.945 mmol, 2.7 equiv), palladium(II) acetate (0.0039 g, 0.018 mmol, 5 mol %), 4,5-bis(diphenylphosphino)-9,9-dimethylxanthene (0.0209 g, 0.036 mmol, 10 mol %) were charged in a nitrogen flushed vial and sealed with a septum. After adding 5 mL *p*-xylene, aniline (**2a**, 0.038 mL, 0.42 mmol, 1.2 equiv) was slowly added dropwise to the reaction mixture, which was then heated up to 125 °C for three hours in a heating block. After cooling to room temperature, the mixture was diluted with water and saturated $NaHCO_3$. The aqueous phase was extracted several times with toluene. The organic phase was washed with water, dried over $MgSO_4$ and concentrated in vacuo. The crude residue was purified by column chromatography on silica gel with heptane/dichloromethane 10:1 giving 38 mg (45%) of **3a** as a colorless solid.

9-(4-Fluorophenyl)-9*H*-carbazole (3f), 2'-bromo-*N*-(4-fluorophenyl)biphenyl-2-amine (7a) and 2'-bromo-*N*-(4-fluorophenyl)biphenyl-3-amine (7b)

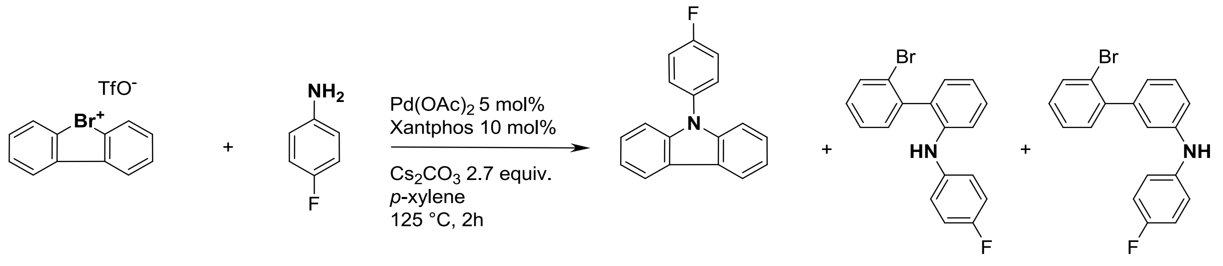

6 **2f** **3f** (25%) **7a** (12%) **7b** (18%)

Using the general procedure with dibenzo[*b*,*d*]bromolium triflate (**6**, 0.133 g, 0.35 mmol, 1.0 equiv) and 4-fluoroaniline (**2f**, 0.04 mL, 0.42 mmol, 1.2 equiv) gave a mixture of 9-(4-fluorophenyl)-9*H*-carbazole (**3f**, 23 mg, 25%) together with the secondary amines **7a** (14 mg, 12%) and **7b** (22 mg, 18%). All three compounds could be separated by column chromatography on silica gel with heptane/dichloromethane 10:1.

III. Characterization data

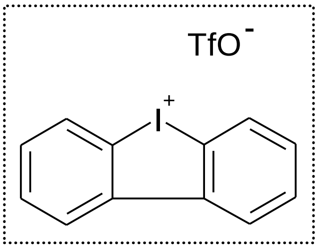

Dibenzo[*b*,*d*]iodol-5-ium trifluoromethanesulfonate (1): off-white powder (3.96 g, 92%); ^{1}H NMR (400 MHz, DMSO-d_6) δ 8.49 (dd, 2H, J_1 = 8.0 Hz, J_2 = 1.4 Hz), 8.22 (dd, 2H, J_1 = 8.3 Hz, J_2 = 0.9 Hz), 7.87 (td, 2H, J_1 = 7.5 Hz, J_2 = 1.0 Hz), 7.72 (td, 2H, J_1 = 7.7 Hz, J_2 = 1.4 Hz); ^{13}C{^{1}H} NMR (100 MHz, DMSO-d_6) δ 141.7, 131.0, 130.7, 130.6, 127.0, 121.6, 120.7 (q, $CF_3SO_3^-$, $^{1}J_{C\text{-}F}$ = 323 Hz); ^{19}F NMR (376.46 MHz, DMSO-d_6) δ –77.7 (s, $CF_3SO_3^-$); IR (neat): 1770, 1759, 1270, 1246, 1234, 1222, 1176, 1157, 1025, 1014, 751, 632 (cm^{-1}); HRMS (APCI) *m/z* calculated for $C_{12}H_8I$: 278.96652, found [M]$^+$: 278.96658.

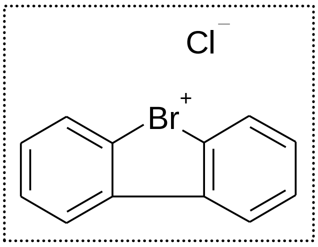

Dibenzo[*b*,*d*]bromolium chloride (5): off-white powder (2.505 g, 52%); ^{1}H NMR (500 MHz, DMSO-d_6) δ 8.81 (dd, 2H, J_1 = 8.6 Hz, J_2 = 0.8 Hz), 8.56 (dd, 2H, J_1 = 7.8 Hz, J_2 = 1.5 Hz), 7.92 (td, 2H, J_1 = 7.6 Hz, J_2 = 0.9 Hz), 7.81 (td, 2H, J_1 = 8.0 Hz, J_2 = 1.6 Hz); ^{13}C{^{1}H} NMR (126 MHz, DMSO-d_6) δ 137.3, 135.2, 131.3, 131.0, 126.0, 125.9; IR (neat): 3053, 2997, 1770, 1759, 1445, 1412, 1245, 741, 638 (cm^{-1}); elemental analysis calculated (%) for $C_{12}H_8BrCl$: C 53.87, H 3.01, found C 54.197, H 2.96.

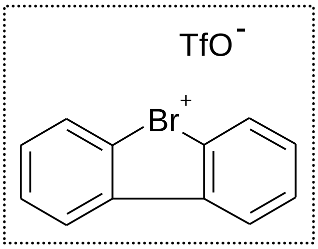

Dibenzo[*b*,*d*]bromolium trifluoromethanesulfonate (6): light gray crystals (1.016 g, quant); ^{1}H NMR (500 MHz, DMSO-d_6) δ 8.60 (dd, 2H, J_1 = 7.9 Hz, J_2 = 1.5 Hz), 8.48 (dd, 2H, J_1 = 8.6 Hz, J_2 = 0.5 Hz), 7.95 (td, 2H, J_1 = 7.6 Hz, J_2 = 0.8 Hz), 7.86 (td, 2H, J_1 = 8.0 Hz, J_2 = 1.6 Hz); ^{13}C{^{1}H} NMR (126 MHz, DMSO-d_6) δ 136.5, 135.3, 131.8, 131.2, 126.3, 125.5, 120.7 (q, $CF_3SO_3^-$, $^{1}J_{C\text{-}F}$ = 323 Hz), the quartet signal arising from the triflate counterion was only observed with low intensity; IR (neat): 1770, 1759, 1247, 1173, 1149, 1057, 1026, 748, 629 (cm^{-1}); HRMS (APCI) *m/z* calculated for $C_{12}H_8Br{*}CF_3O_3S$: 379.93296, found [M]$^+$: 379.93268; HRMS (APCI) *m/z* calculated for $C_{12}H_8Br$: 230.98094, found [M]$^+$: 230.98042.

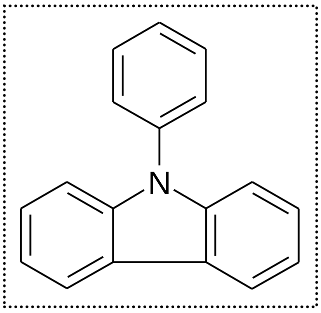

9-Phenyl-9*H*-carbazole (3a): colorless solid (38 mg, 45%); eluent: heptane/dichloromethane v/v 10:1; ^{1}H NMR (400 MHz, $CDCl_3$) δ 8.16 (d, 2H, J = 7.8 Hz), 7.63-7.56 (m, 4H), 7.47 (td, 1H, J_1 = 7.0 Hz, J_2=1.7 Hz), 7.41 (d, 4H, J = 4.0 Hz), 7.33-7.27 (m, 2H); $^{13}C\{^{1}H\}$ NMR (100 MHz, $CDCl_3$) δ 141.1, 137.9, 130.0, 127.6, 127.3, 126.1, 123.5, 120.4, 120.0, 109.9; GC–MS (EI) *m/z* calculated for $C_{18}H_{13}N$: 243.105, found $[M]^{+}$: 243.105. The spectral data were in good agreement with those reported in the literature [6].

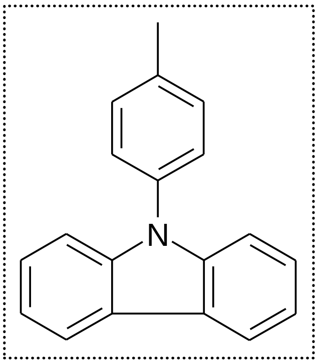

9-(*p*-Tolyl)-9*H*-carbazole (3b): colorless solid (51 mg, 45%); eluent: heptane/dichloromethane v/v 10:1; ^{1}H NMR (500 MHz, $CDCl_3$) δ 8.15 (d, 2H, J = 7.8 Hz), 7.45-7.37 (m, 8H), 7.28 (td, 2H, J_1 = 7.1 Hz, J_2 = 1.7 Hz), 2.50 (s, 3H); $^{13}C\{^{1}H\}$ NMR (126 MHz, $CDCl_3$) δ 141.3, 137.5, 135.2, 130.6, 127.2, 126.0, 123.4, 120.4, 119.9, 109.9, 21.4; GC–MS (EI) *m/z* calculated for $C_{19}H_{15}N$: 257.1, found $[M]^{+}$: 257.5. The ^{13}C NMR is in good agreement with those data reported in the literature [7].

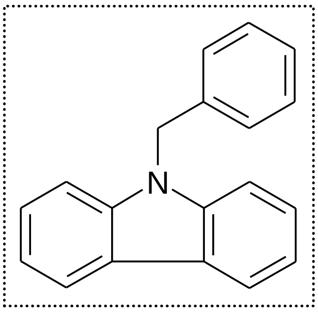

9-Benzyl-9*H*-carbazole (3c): colorless solid (37 mg, 41%); eluent: heptane/dichloromethane v/v 10:1 → 4:1; ^{1}H NMR (500 MHz, $CDCl_3$) δ 8.14 (d, 2H, J = 7.7 Hz), 7.43 (td, 2H, J_1 = 7.6 Hz, J_2 = 1.2 Hz), 7.37 (d, 2H, J = 8.2 Hz), 7.28-7.23 (m, 5H), 7.15 (d, 2H, J = 7.4 Hz), 5.53 (s, 2H); $^{13}C\{^{1}H\}$ NMR (126 MHz, $CDCl_3$) δ 140.9, 137.4, 128.9, 127.6, 126.6, 126.0, 123.2, 120.5, 119.4, 109.1, 46.8; GC–MS (EI) *m/z* calculated for $C_{19}H_{15}N$: 257.1, found $[M]^{+}$: 257.4. The spectral data were in good agreement with those reported in the literature [7].

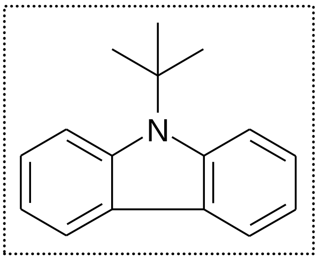

9-(*tert*-Butyl)-9*H*-carbazole (3d): colorless solid (5 mg, 3%); eluent: heptane/dichloromethane v/v 10:1; ^{1}H NMR (500 MHz, $CDCl_3$) δ 8.10 (dd, 2H, J_1 = 7.8 Hz, J_2 = 0.7 Hz), 7.86 (d, 2H, J = 8.6 Hz), 7.37 (td, 2H, J_1 = 7.9 Hz, J_2 = 1.4 Hz), 7.20 (t, 2H, J_1 = 7.5 Hz), 2.01 (s, 9H); ^{13}C{^{1}H} NMR (126 MHz, $CDCl_3$) δ 140.6, 125.2, 124.6, 120.0, 118.6, 113.9, 59.2, 31.2; GC–MS (EI) *m/z* calculated for $C_{16}H_{17}N$: 223.1, found $[M]^+$: 223.2. The spectral data were in good agreement with those reported in the literature [8].

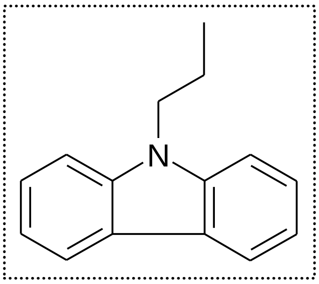

9-Propyl-9*H*-carbazole (3e): colorless oil (58 mg, 79%); eluent: heptane/dichloromethane v/v 10:1; ^{1}H NMR (500 MHz, $CDCl_3$) δ 8.11 (d, 2H, J = 7.8 Hz), 7.46 (td, 2H, J_1 = 7.6 Hz, J_2 = 1.1 Hz), 7.42 (d, 2H, J = 8.1 Hz), 7.23 (td, 2H, J_1 = 7.4 Hz, J_2 = 1.0 Hz), 4.29 (t, 2H, J = 7.2 Hz), 1.93 (sext, 2H, J = 7.3 Hz), 0.99 (t, 3H, J = 7.5 Hz); ^{13}C{^{1}H} NMR (126 MHz, $CDCl_3$) δ 140.7, 125.7, 123.0, 120.5, 118.9, 108.8, 44.8, 22.5, 12.0; GC–MS (EI) *m/z* calculated for $C_{15}H_{15}N$: 209.1, found $[M]^+$: 209.4. The spectral data were in good agreement with those reported in the literature [9].

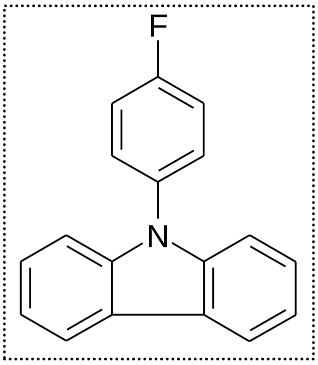

9-(4-Fluorophenyl)-9*H*-carbazole (3f): colorless solid (65 mg, 71%); eluent: heptane/dichloromethane v/v 10:1; ^{1}H NMR (500 MHz, $CDCl_3$) δ 8.15 (d, 2H, J = 7.8 Hz), 7.54 (d, 1H, J = 4.9 Hz), 7.52 (d, 1H, J = 4.9 Hz), 7.42 (td, 2H, J_1 = 7.7 Hz, J_2 = 1.2 Hz), 7.34-7.28 (m, 6H); ^{13}C{^{1}H} NMR (126 MHz, $CDCl_3$) δ 161.8 (d, $^{1}J_{C\text{-}F}$ = 248 Hz), 141.2, 133.8 (d, $^{4}J_{C\text{-}F}$ = 3 Hz), 129.2 (d, $^{3}J_{C\text{-}F}$ = 9 Hz), 126.2, 123.5, 120.5, 120.2, 117.0 (d, $^{2}J_{C\text{-}F}$ = 23 Hz), 109.7; GC–MS (EI) *m/z* calculated for $C_{18}H_{12}FN$: 261.1, found $[M]^+$: 261.5. The ^{13}C NMR is in good agreement with those data reported in the literature [7], whereas the ^{1}H NMR differs slightly.

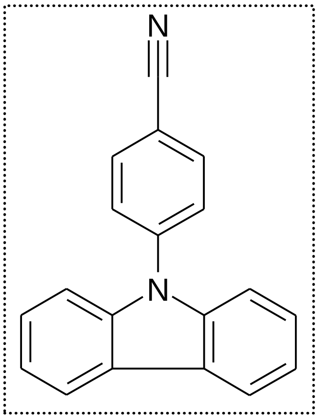

4-(9*H*-Carbazol-9-yl)benzonitrile (3g): pale yellow crystals (50 mg, 53%); eluent: heptane/dichloromethane v/v 2:1 → 1:1; ^{1}H NMR (500 MHz, $CDCl_3$) δ 8.15 (d, 2H, J = 7.70 Hz), 7.91 (d, 2H, J = 8.5 Hz), 7.75 (d, 2H, J = 8.5 Hz), 7.47-7.42 (m, 4H), 7.34 (td, 2H, J_1 = 7.0 Hz, J_2 = 1.9 Hz); ^{13}C{^{1}H} NMR (126 MHz, $CDCl_3$) δ 142.3, 140.1, 134.1, 127.3, 126.5, 124.2, 121.2, 120.7, 118.5, 110.7, 109.7; GC–MS (EI) *m/z* calculated for $C_{19}H_{12}N_2$: 268.1, found $[M]^+$: 268.6. The ^{13}C NMR is in good agreement with those data reported in the literature [10], whereas the ^{1}H NMR differs slightly.

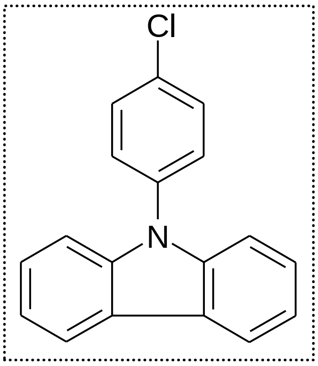

9-(4-Chlorophenyl)-9*H*-carbazole (3h): pale yellow solid (53 mg, 55%); eluent: heptane/dichloromethane v/v 10:1; ^{1}H NMR (500 MHz, $CDCl_3$) δ 8.15 (d, 2H, J = 7.8 Hz), 7.58 (d, 2H, J = 8.7 Hz), 7.51 (d, 2H, J = 8.6 Hz), 7.42 (td, 2H, J_1 = 7.6 Hz, J_2 = 1.1 Hz), 7.37 (d, 2H, J = 8.1 Hz), 7.30 (td, 2H, J_1 = 7.4 Hz, J_2 = 1.0 Hz); ^{13}C{^{1}H} NMR (126 MHz, $CDCl_3$) δ 140.9, 136.5, 133.2, 130.3, 128.6, 126.2, 123.6, 120.5, 120.3, 109.7; GC–MS (EI) *m/z* calculated for $C_{18}H_{12}ClN$: 277.1, found $[M]^+$: 277.5. The spectral data were in general in good agreement with those reported in the literature [7].

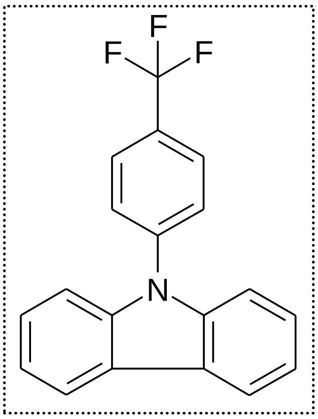

9-(4-(Trifluoromethyl)phenyl)-9*H*-carbazole (3i): colorless solid (66 mg, 61%); eluent: heptane/dichloromethane v/v 10:1; ^{1}H NMR (500 MHz, $CDCl_3$) δ 8.16 (d, 2H, J = 7.8 Hz), 7.89 (d, 2H, J = 8.0 Hz), 7.73 (d, 2H, J = 8.3 Hz), 7.45-7.41 (m, 4H), 7.35-7.30 (m, 2H); ^{13}C{^{1}H} NMR (126 MHz, $CDCl_3$) δ 141.3, 140.5, 129.4 (q, $^{2}J_{C\text{-}F}$ = 33 Hz), 127.3 (q, $^{3}J_{C\text{-}F}$ = 4 Hz), 127.2, 126.4, 124.1 (q, $^{1}J_{C\text{-}F}$ = 273 Hz), 123.9, 120.7, 120.6, 109.7; GC–MS (EI) *m/z* calculated for $C_{19}H_{12}F_3N$: 311.1, found $[M]^+$: 311.5. The spectral data were in good agreement with those reported in the literature [7].

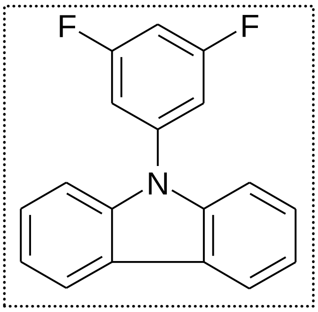

9-(3,5-Difluorophenyl)-9H-carbazole (3j): colorless solid (53 mg, 54%); eluent: heptane/dichloromethane v/v 10:1; ^{1}H NMR (500 MHz, $CDCl_3$) δ 8.14 (d, 2H, J = 7.8 Hz), 7.48 (d, 2H, J = 8.1 Hz), 7.45 (td, 2H, J_1 = 7.5 Hz, J_2 = 1.2 Hz), 7.33 (td, 2H, J_1 = 7.3 Hz, J_2 = 1.3 Hz), 7.17 (dd, 2H, J_1 = 7.8 Hz, J_2 = 2.3 Hz), 6.93 (ttt, 1H, J_1 = 8.9 Hz, J_2 = 2.4 Hz); ^{13}C{^{1}H} NMR (126 MHz, $CDCl_3$) δ 163.9 (dd, $^1J_{C\text{-}F}$ = 250 Hz, $^3J_{C\text{-}F}$ = 15 Hz), 140.3, 140.3, 126.5, 123.9, 120.9, 120.6, 110.3 (dd, $^2J_{C\text{-}F}$ = 20 Hz, $^4J_{C\text{-}F}$ = 7 Hz), 109.8, 103.1 (t, $^2J_{C\text{-}F}$ = 25 Hz), a $^3J_{C\text{-}F}$ coupling from the quaternary carbon attached to the nitrogen atom in the aniline ring was not observed; IR (neat): 1608, 1578, 1492, 1444, 1334, 1310, 1275, 1226, 1155, 1115, 1019, 990, 915, 862, 851, 823, 743, 721, 691, 670, 640, 615 (cm^{-1}); HRMS (APCI) m/z calculated for $C_{18}H_{11}F_2N$: 279.08596, found $[M+H]^+$: 280.09283.

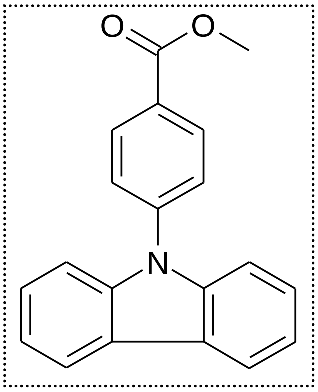

Methyl 4-(9H-carbazol-9-yl)benzoate (3k): light brown solid (65 mg, 62%); eluent: heptane/dichloromethane v/v 1:1 → 1:2; ^{1}H NMR (500 MHz, $CDCl_3$) δ 8.29 (d, 2H, J = 8.7 Hz), 8.15 (d, 2H, J = 7.8 Hz), 7.69 (d, 2H, J = 8.5 Hz), 7.47 (d, 2H, J = 8.2 Hz), 7.43 (td, 2H, J_1 = 7.6 Hz, J_2 = 1.2 Hz), 7.32 (td, 2H, J_1 = 7.4 Hz, J_2 = 1.2 Hz), 4.00 (s, 3H); ^{13}C{^{1}H} NMR (126 MHz, $CDCl_3$) δ 166.5, 142.2, 140.5, 131.5, 128.9, 126.6, 126.3, 124.0, 120.7, 120.6, 109.9, 52.5; GC–MS (EI) m/z calculated for $C_{20}H_{15}NO_2$: 301.1, found $[M]^+$: 301.6. The proton NMR data were in good agreement with those reported the literature [11,12]. ^{13}C NMR data for this compound were so far not reported.

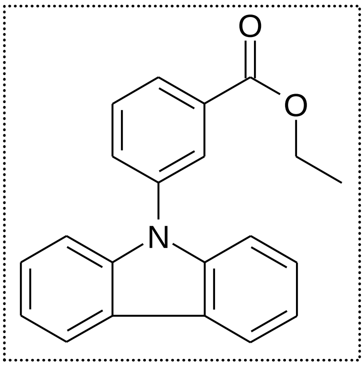

Ethyl 3-(9H-carbazol-9-yl)benzoate (3l): light brown solid (71 mg, 64%); eluent: heptane/dichloromethane v/v 2:1 → 1:1; ^{1}H NMR (500 MHz, $CDCl_3$) δ 8.26 (t, 1H, J = 1.6 Hz), 8.16 (d, 3H, J = 7.9 Hz), 7.77 (m, 1H), 7.69 (t, 1H, J = 7.8 Hz), 7.42 (td, 2H, J_1 = 7.6 Hz, J_2 = 1.1 Hz), 7.39 (d, 2H, J = 8.1 Hz), 7.31 (td, 2H, J_1 = 7.3 Hz, J_2 = 1.1 Hz), 4.42 (q, 2H, J = 7.2 Hz), 1.40 (t, 3H, J = 7.2 Hz); ^{13}C{^{1}H} NMR (126 MHz, $CDCl_3$) δ 166.0, 140.9, 138.2, 132.8, 131.6, 130.1, 128.7, 128.4, 126.2, 123.7, 120.5, 120.4, 109.7, 61.5, 14.5; IR

(neat): 1727, 1587, 1496, 1478, 1447, 1363, 1336, 1313, 1291, 1255, 1237, 1226, 1177, 1120, 1099, 1078, 1021, 1002, 919, 748, 718, 689, 628 (cm^{-1}); GC–MS (EI) *m/z* calculated for $C_{21}H_{17}NO_2$: 315.1, found $[M]^+$: 315.4; elemental analysis calculated (%) for $C_{21}H_{17}NO_2$: C 79.98, H 5.43, N 4.44, O 10.15 found C 79.31, H 5.27, N 4.10.

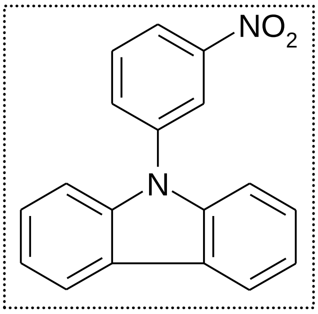

9-(3-Nitrophenyl)-9*H*-carbazole (3m): yellow solid (61 mg, 61%); eluent: heptane/dichloromethane v/v 5:1 → 2:1; ^{1}H NMR (500 MHz, $CDCl_3$) δ 8.49 (t, 1H, J = 2.1 Hz), 8.32 (ddd, 1H, J_1 = 8.3 Hz, J_2 = 2.2 Hz, J_3 = 1.0 Hz), 8.16 (d, 2H, J = 7.8 Hz), 7.96 (ddd, 1H, J_1 = 7.9 Hz, J_2 = 2.1 Hz, J_3 = 1.0 Hz), 7.81 (t, 1H, J = 8.1 Hz), 7.45 (td, 2H, J_1 = 7.5 Hz, J_2 = 1.2 Hz), 7.42 (d, 2H, J = 7.90 Hz), 7.35 (td, 2H, J_1 = 7.3 Hz, J_2 = 1.3 Hz); $^{13}C\{^1H\}$ NMR (126 MHz, $CDCl_3$) δ 149.6, 140.4, 139.4, 133.0, 131.0, 126.6, 124.0, 122.1, 122.1, 121.1, 120.8, 109.5; GC–MS (EI) *m/z* calculated for $C_{18}H_{12}N_2O_2$: 288.1, found $[M]^+$: 288.5. The spectral data were in general in good agreement with those reported in the literature [7].

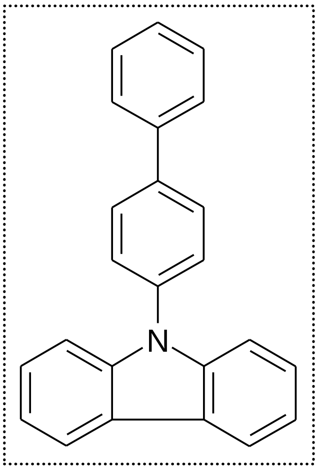

9-([1,1'-Biphenyl]-4-yl)-9*H*-carbazole (3n): colorless solid (45 mg, 40%); eluent: heptane/dichloromethane v/v 10:1; ^{1}H NMR (500 MHz, $CDCl_3$) δ 8.17 (d, 2H, J = 7.8 Hz), 7.83 (d, 2H, J = 8.5 Hz), 7.70 (dd, 2H, J_1 = 8.3 Hz, J_2 = 1.3 Hz), 7.65 (d, 2H, J = 8.5 Hz), 7.52 (t, 2H, J = 7.1 Hz), 7.48 (d, 2H, J = 8.2 Hz), 7.45-7.40 (m, 3H), 7.31 (td, 2H, J_1 = 7.4 Hz, J_2 = 1.1 Hz); $^{13}C\{^1H\}$ NMR (126 MHz, $CDCl_3$) δ 141.1, 140.5, 140.5, 137.0, 129.1, 128.7, 127.8, 127.5, 127.3, 126.1, 123.6, 120.5, 120.1, 110.0; GC–MS (EI) *m/z* calculated for $C_{24}H_{17}N$: 319.1, found $[M]^+$: 319.7. The spectral data were in good agreement with those reported in the literature [13].

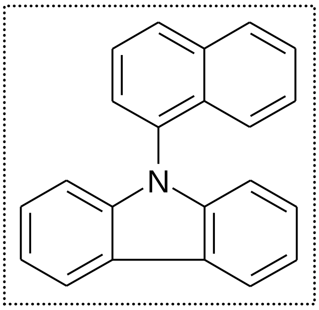

9-(Naphthalen-1-yl)-9*H*-carbazole (3o): pale yellow solid (24 mg, 23%), eluent: heptane/dichloromethane v/v 10:1; ^{1}H NMR (500 MHz, $CDCl_3$) δ 8.22 (d, 2H, J = 7.7 Hz), 8.05 (d, 1H, J = 7.9 Hz), 8.02 (d, 1H, J = 8.3 Hz), 7.69-7.63 (m, 2H), 7.54 (td, 1H, J_1 = 7.5 Hz, J_2 = 1.2 Hz), 7.36-7.33 (m, 3H), 7.31 (dd, 2H, J_1 = 7.7 Hz, J_2 = 1.1 Hz), 7.29-7.28 (m, 1H), 7.01 (d, 2H, J = 8.0 Hz); ^{13}C{^{1}H} NMR (125.8 MHz, $CDCl_3$) δ 142.4, 135.0, 134.2, 131.1, 129.2, 128.6, 127.1, 126.9, 126.9, 126.1, 126.1, 123.7, 123.4, 120.4, 119.9, 110.4; GC–MS (EI) *m/z* calculated for $C_{22}H_{15}N$: 293.1, found $[M]^+$: 293.5. The ^{13}C NMR is in good agreement with those data reported in the literature [7], whereas the ^{1}H NMR differs.

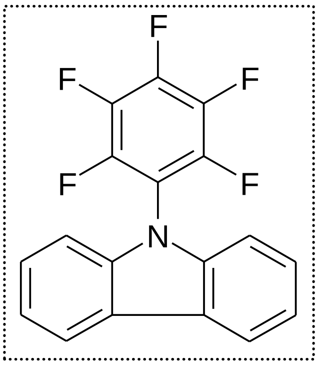

9-(Perfluorophenyl)-9*H*-carbazole (3p): colorless solid (43 mg, 37%), eluent: heptane/dichloromethane v/v 10:1; ^{1}H NMR (500 MHz, $CDCl_3$) δ 8.14 (d, 2H, J = 7.8 Hz), 7.46 (td, 2H, J_1 = 7.7 Hz, J_2 = 1.2 Hz), 7.36 (td, 2H, J_1 = 7.5 Hz, J_2 = 0.8 Hz), 7.12 (d, 2H, J = 8.2 Hz); ^{13}C{^{1}H, ^{19}F} NMR (100 MHz, $CDCl_3$) δ 144.9, 141.6, 140.2, 138.5, 126.7, 124.3, 121.4, 120.8, 112.7, 109.6; ^{19}F NMR (376.46 MHz, $CDCl_3$) δ -142.5 (d, $^{3}J_{F\text{-}F}$ = 24 Hz), -153.2 (t, $^{3}J_{F\text{-}F =}$ 22 Hz), -160.4 (td, $^{3}J_{F\text{-}F}$ = 21 Hz, $^{4}J_{F\text{-}F}$ = 4 Hz); IR (neat): 1770, 1759, 1508, 1478, 1453, 1444, 1315, 1246, 1228, 995, 814, 744, 722, 662, 616 (cm^{-1}); HRMS (APCI) *m/z* calculated for $C_{18}H_8F_5N$: 333.05769, found $[M]^+$: 333.05692.

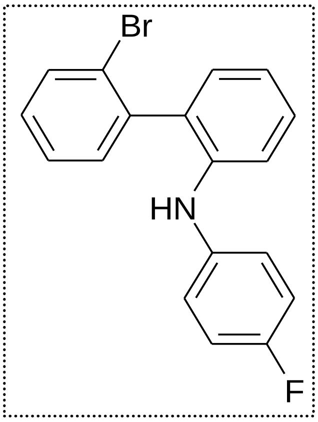

2’-Bromo-*N*-(4-fluorophenyl)biphenyl-2-amine (7a): light brown solid (14 mg, 12%); R_f = 0.52 (heptane/toluene 1:1) [14]; ^{1}H NMR (500 MHz, $CDCl_3$) δ 7.70 (dd, 1H, J_1 = 8.0 Hz, J_2 = 1.1 Hz), 7.40 (td, 1H, J_1 = 7.4 Hz, J_2 = 1.2 Hz), 7.35 (dd, 1H, J_1 = 7.6 Hz, J_2 = 1.9 Hz), 7.29-7.27 (m, 1H), 7.25-7.23 (m, 1H), 7.16 (dd, 1H, J_1 = 8.3 Hz, J_2 = 0.9 Hz), 7.13 (dd, 1H, J_1 = 7.6 Hz, J_2 = 1.6 Hz), 7.03 (d, 1H, J = 4.8 Hz), 7.02 (d, 1H, J = 4.8 Hz), 6.97-6.93 (m, 3H), 5.15 (br s, 1H); ^{13}C{^{1}H} NMR (125.8 MHz, $CDCl_3$) δ 158.6 (d, $^{1}J_{C\text{-}F}$ = 241 Hz), 141.9, 139.9, 138.9 (d, $^{4}J_{C\text{-}F}$ = 3 Hz), 133.3, 132.1, 130.8, 129.9, 129.6, 129.1, 128.1, 124.7, 122.2 (d, $^{3}J_{C\text{-}F}$ = 8 Hz), 120.1, 116.0 (d, $^{2}J_{C\text{-}F}$ = 23 Hz), 115.6; IR (neat): 3397, 1769,

1760, 1578, 1504, 1470, 1443, 1422, 1308, 1247, 1213, 1159, 1096, 1061, 1004, 820, 772, 755, 662, 614 (cm^{-1}); HRMS (APCI) *m/z* calculated for $C_{18}H_{13}BrFN$: 341.02154, found $[M+H]^+$: 342.02877.

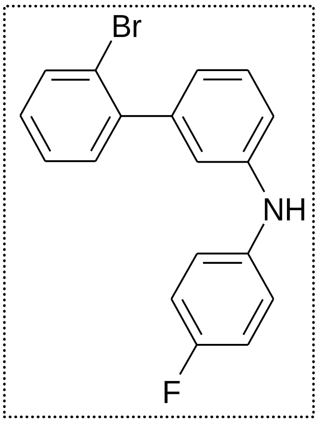

2'-Bromo-*N*-(4-fluorophenyl)biphenyl-3-amine (7b): brown oil (22 mg, 18 %); R_f = 0.40 (heptane/toluene 1:1); ^1H NMR (500 MHz, $CDCl_3$) δ 7.65 (d, 1H, *J* = 8.0 Hz), 7.36-7.32 (m, 2H), 7.30 (t, 1H, *J* = 7.7 Hz), 7.21-7.17(m, 1H), 7.11 (d, 1H, *J* = 4.7 Hz), 7.09 (d, 1H, *J* = 4.7 Hz), 7.02-6.96 (m, 4H), 6.92 (d, 1H, *J* = 7.6 Hz), 5.64 (br s, 1H); $^{13}C\{^1H\}$ NMR (125.8 MHz, $CDCl_3$) δ 158.4 (d, $^1J_{C-F}$ = 241 Hz), 143.8, 142.7, 142.5, 138.9 (d, $^4J_{C-F}$ = 3 Hz), 133.2, 131.3, 129.2, 128.9, 127.5, 122.7, 121.7, 121.0 (d, $^3J_{C-F}$ = 8 Hz), 117.8, 116.1 (d, $^2J_{C-F}$ = 23 Hz), 116.1; IR (neat): 3401, 1770, 1759, 1604, 1583, 1503, 1464, 1324, 1241, 1214, 1021, 824, 786, 752, 698, 662 (cm^{-1}); HRMS (APCI) *m/z* calculated for $C_{18}H_{13}BrFN$: 341.02154, found $[M+H]^+$: 342.02893.

IV. References

1. Poriel, C.; Ferrand, Y.; Juillard, S.; Le Maux, P.; Simonneaux, G. *Tetrahedron* **2004,** *60*, 145-158. doi:10.1016/j.tet.2003.10.080

2. Pan, X.; Wilcox, C. S. *J. Org. Chem.* **2010,** *75*, 6445-6451. doi:10.1021/jo101137r

3. Sandin, R. B.; Hay, A. S. *J. Am. Chem. Soc.* **1952,** *74*, 274-275. doi:10.1021/ja01121a524

4. Bielawski, M.; Olofsson, B. *Chem. Commun.* **2007**, 2521-2523. doi:10.1039/B701864A

5. Bielawski, M.; Zhu, M.; Olofsson, B. *Adv. Synth. Catal.* **2007,** *349*, 2610-2618. doi:10.1002/adsc.200700373

6. Xu, Z.-L.; Li, H.-X.; Ren, Z.-G.; Du, W.-Y.; Xu, W.-C.; Lang, J.-P. *Tetrahedron* **2011,** *67*, 5282-5288. doi:10.1016/j.tet.2011.05.025

7. Zhou, Y.; Verkade, J. G. *Adv. Synth. Catal.* **2010,** *352*, 616-620. doi:10.1002/adsc.200900846

8. Jordan-Hore, J. A.; Johansson, C. C. C.; Gulias, M.; Beck, E. M.; Gaunt, M. J. *J. Am. Chem. Soc.* **2008,** *130*, 16184-16186. doi:10.1021/ja806543s

9. Milen, M.; Grün, A.; Bálint, E.; Dancsó, A.; Keglevich, G. *Synthetic Commun.* **2010,** *40*, 2291-2301. doi:10.1080/00397910903226166

10. Suzuki, K.; Hori, Y.; Kobayashi, T. *Adv. Synth. Catal.* **2008,** *350*, 652-656. doi:10.1002/adsc.200700543

11. Smith, C. J.; Tsang, M. W. S.; Holmes, A. B.; Danheiser, R. L.; Tester, J. W. *Org. Biomol. Chem.* **2005,** *3*, 3767-3781. doi:10.1039/B509345G

12. Kato, Y.; Conn, M. M.; Rebek, J., Jr. *J. Am. Chem. Soc.* **1994,** *116*, 3279-3284. doi:10.1021/ja00087a013

13. Maligres, P. E.; Krska, S. W.; Dormer, P. G. *J. Org. Chem.* **2012,** *77*, 7646-7651. doi:10.1021/jo300905w

14. Kitawaki, T.; Hayashi, Y.; Ueno, A.; Chida, N. *Tetrahedron* **2006,** *62*, 6792-6801. doi:10.1016/j.tet.2006.04.087

V. Copies of ^{1}H NMR, ^{13}C NMR, and ^{19}F NMR spectra

Dibenzo[*b*,*d*]iodol-5-ium trifluoromethanesulfonate (1)

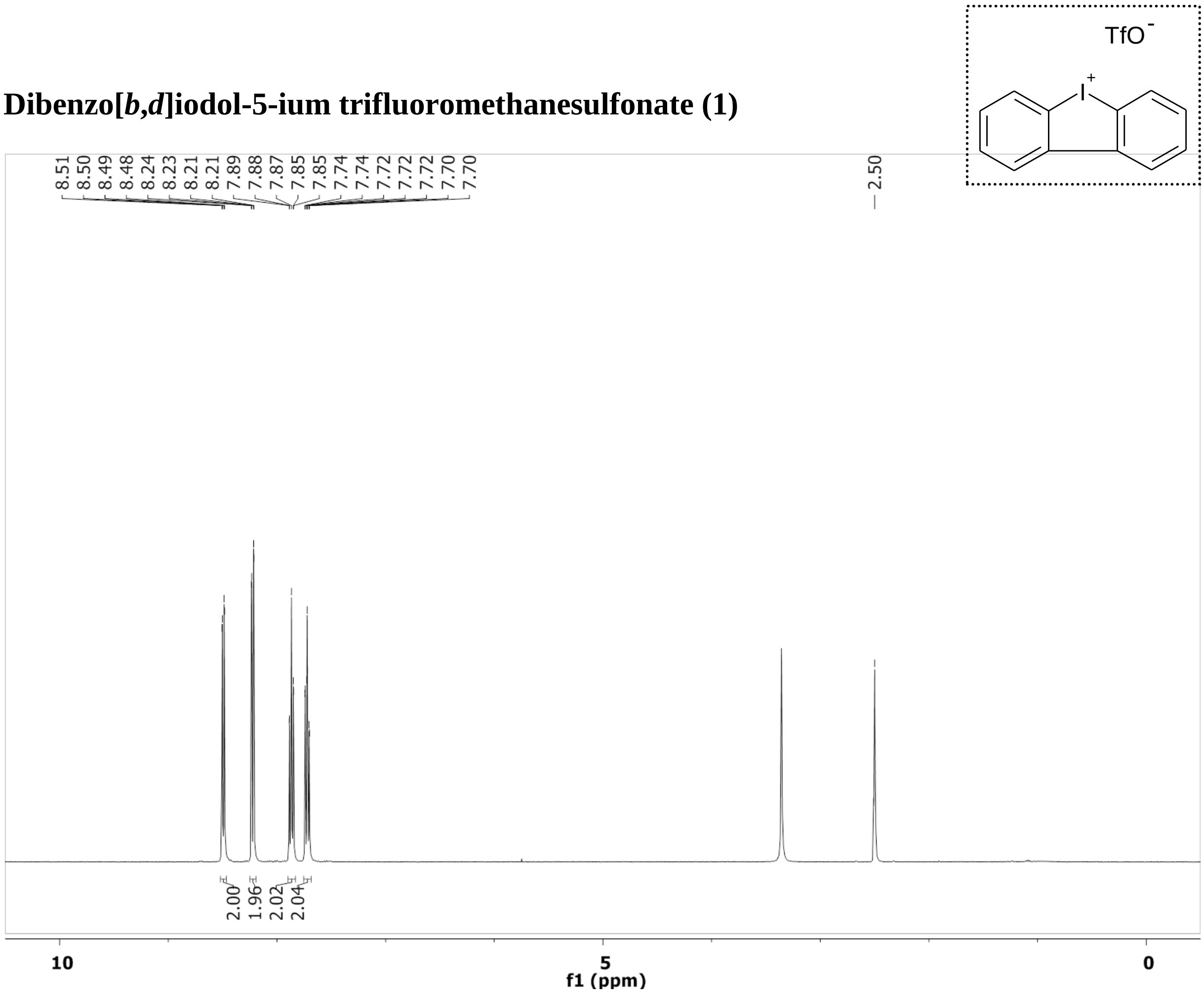

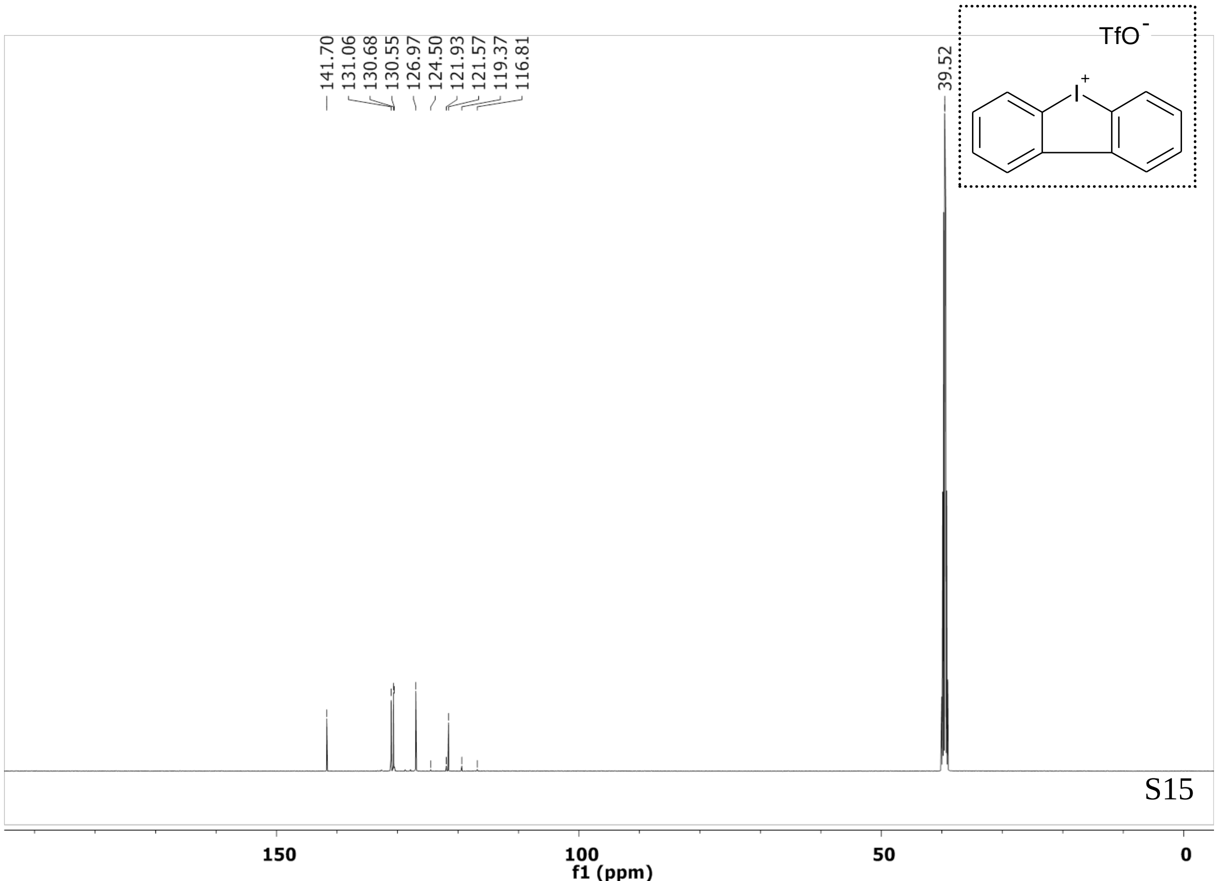

Dibenzo[*b*,*d*]iodol-5-ium trifluoromethanesulfonate (1)

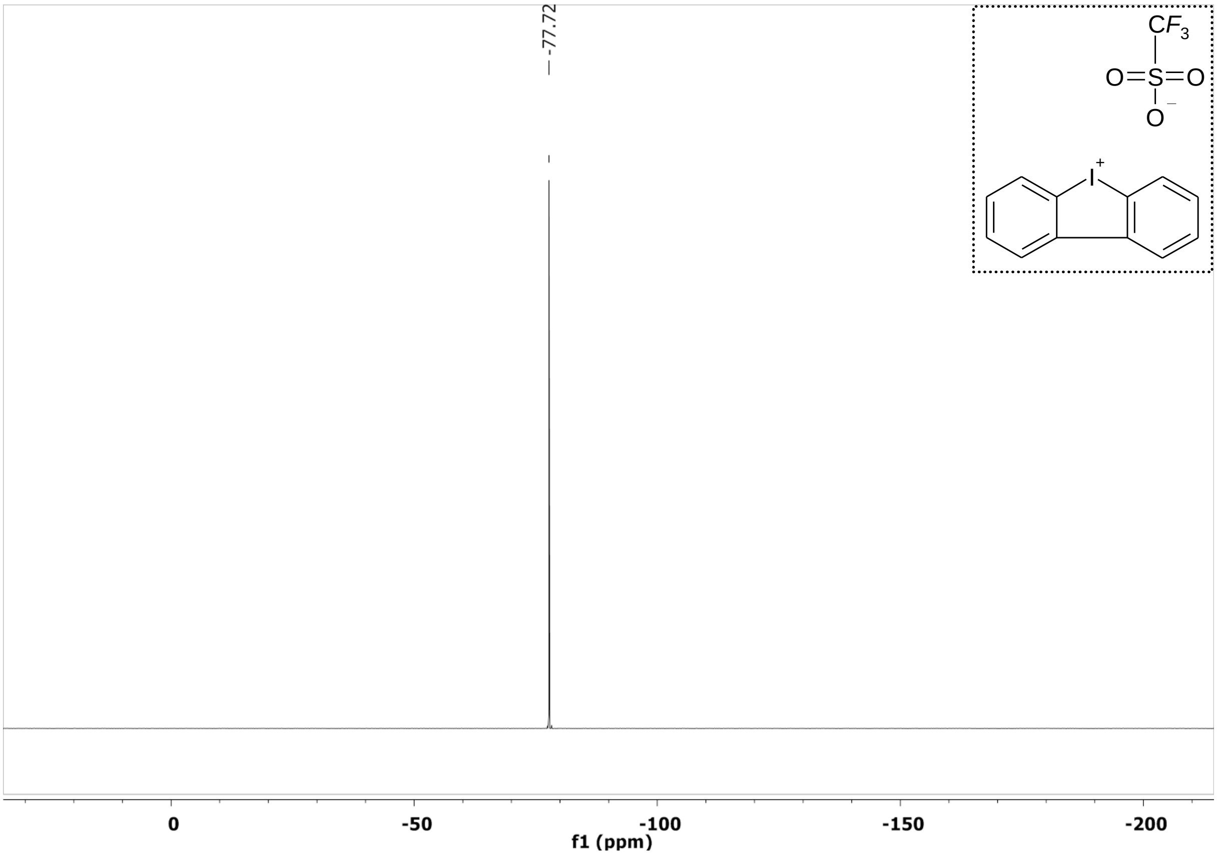

Dibenzo[*b*,*d*]bromolium chloride (5)

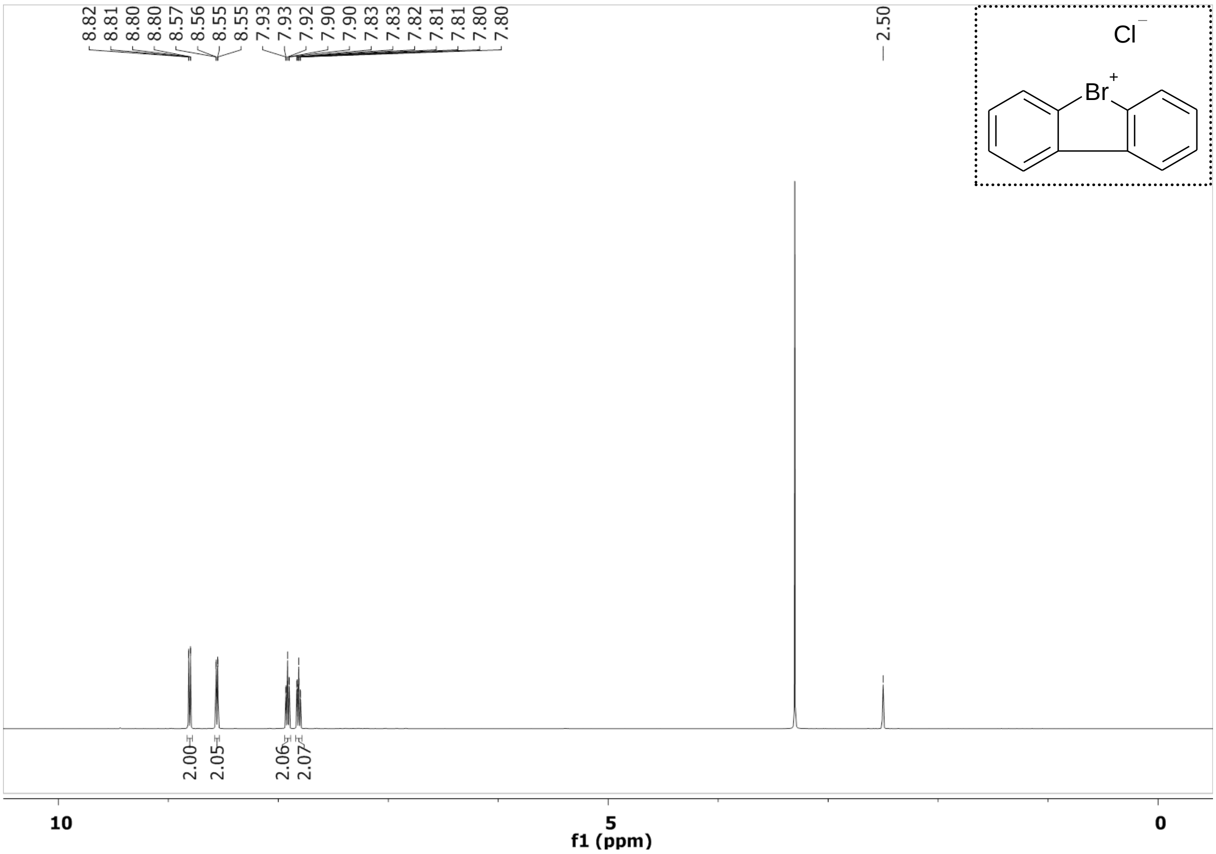

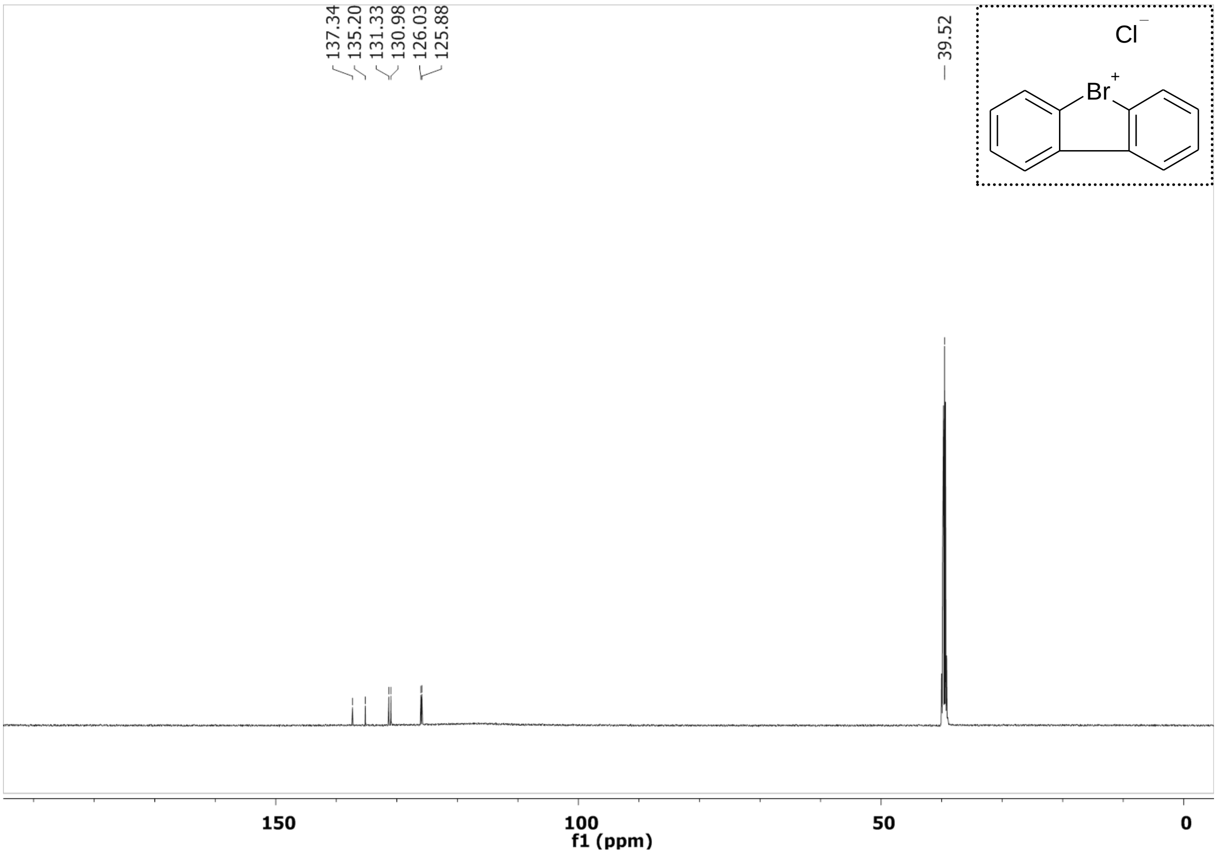

Dibenzo[*b*,*d*]bromolium trifluoromethanesulfonate (6)

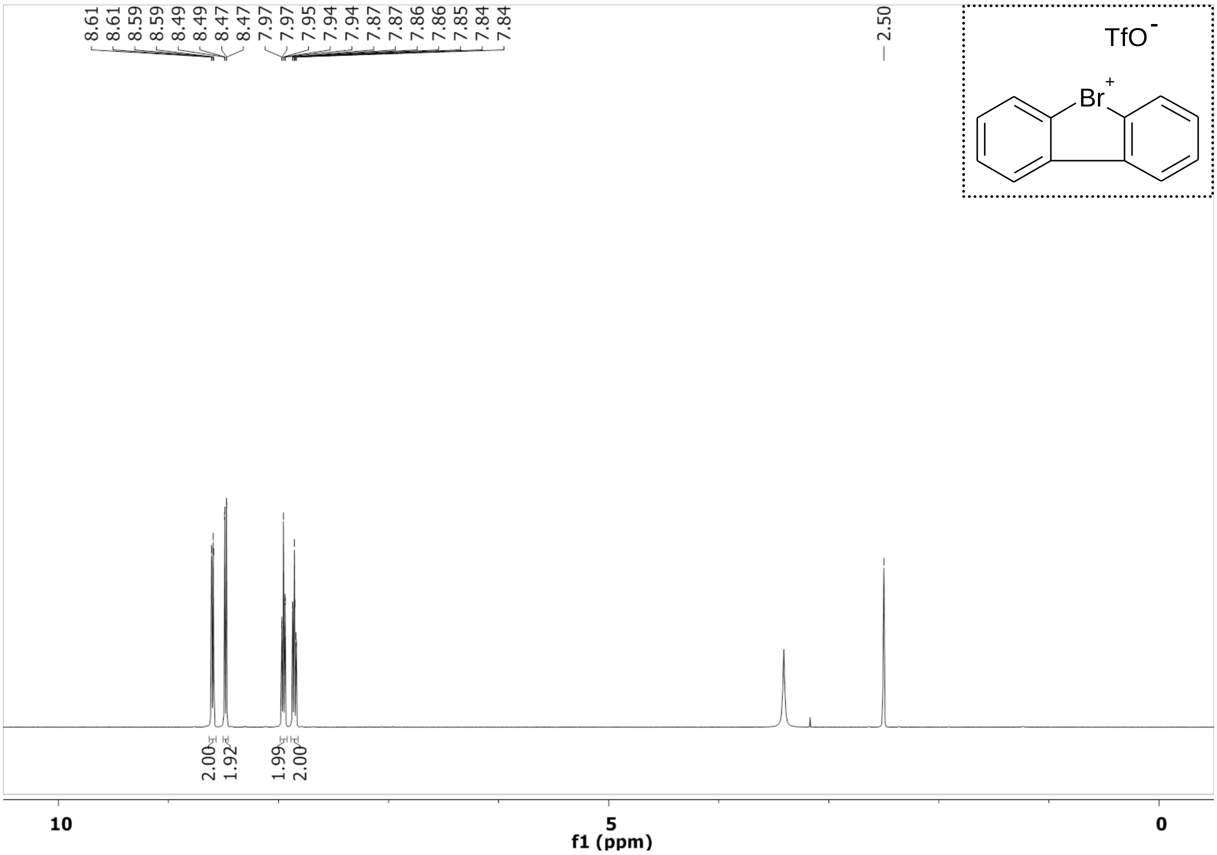

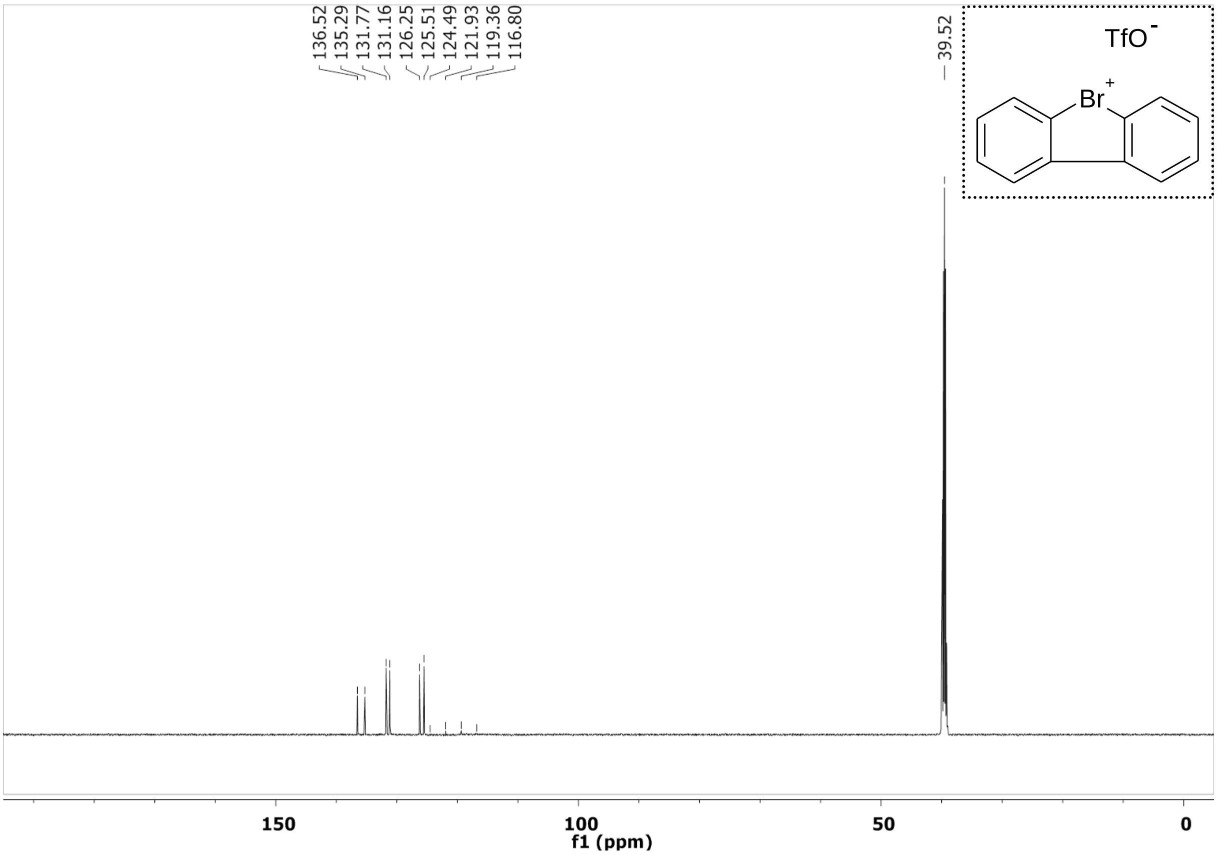

9-Phenyl-9*H*-carbazole (3a)

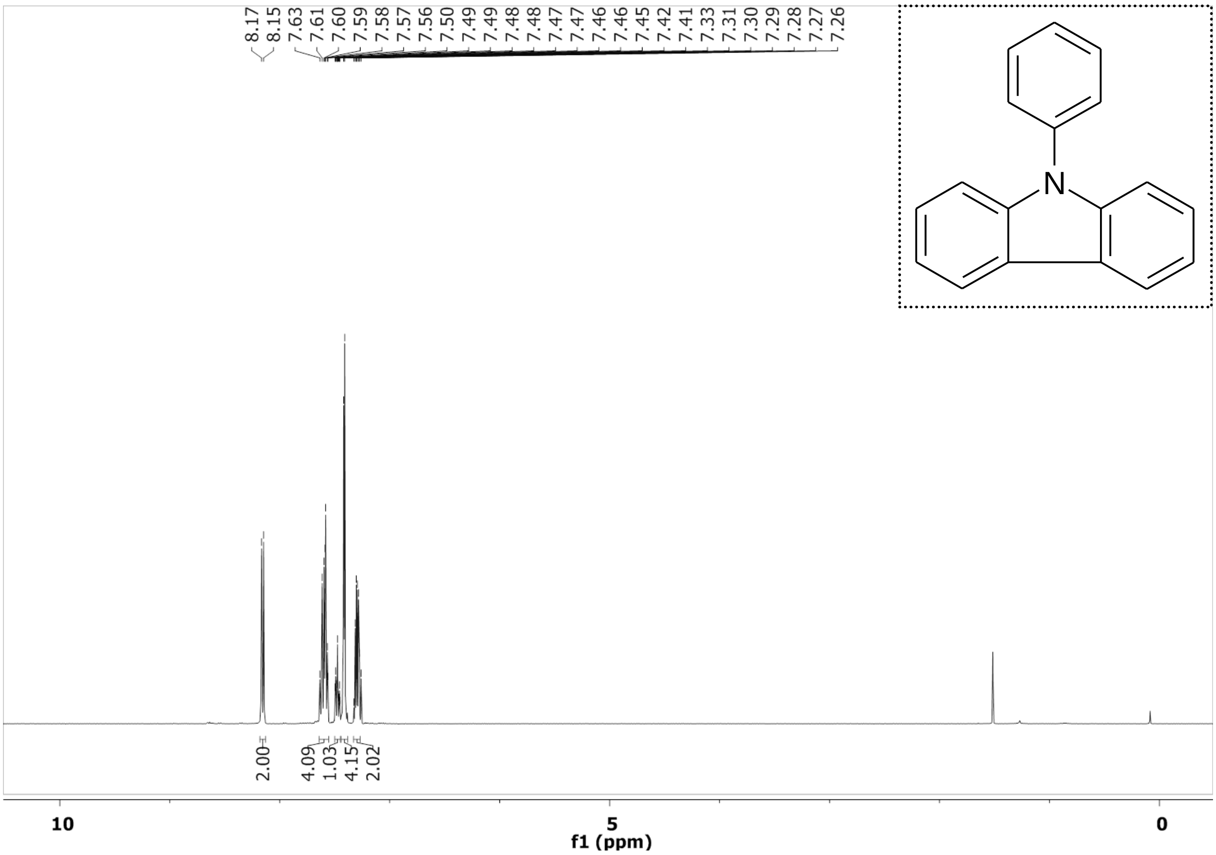

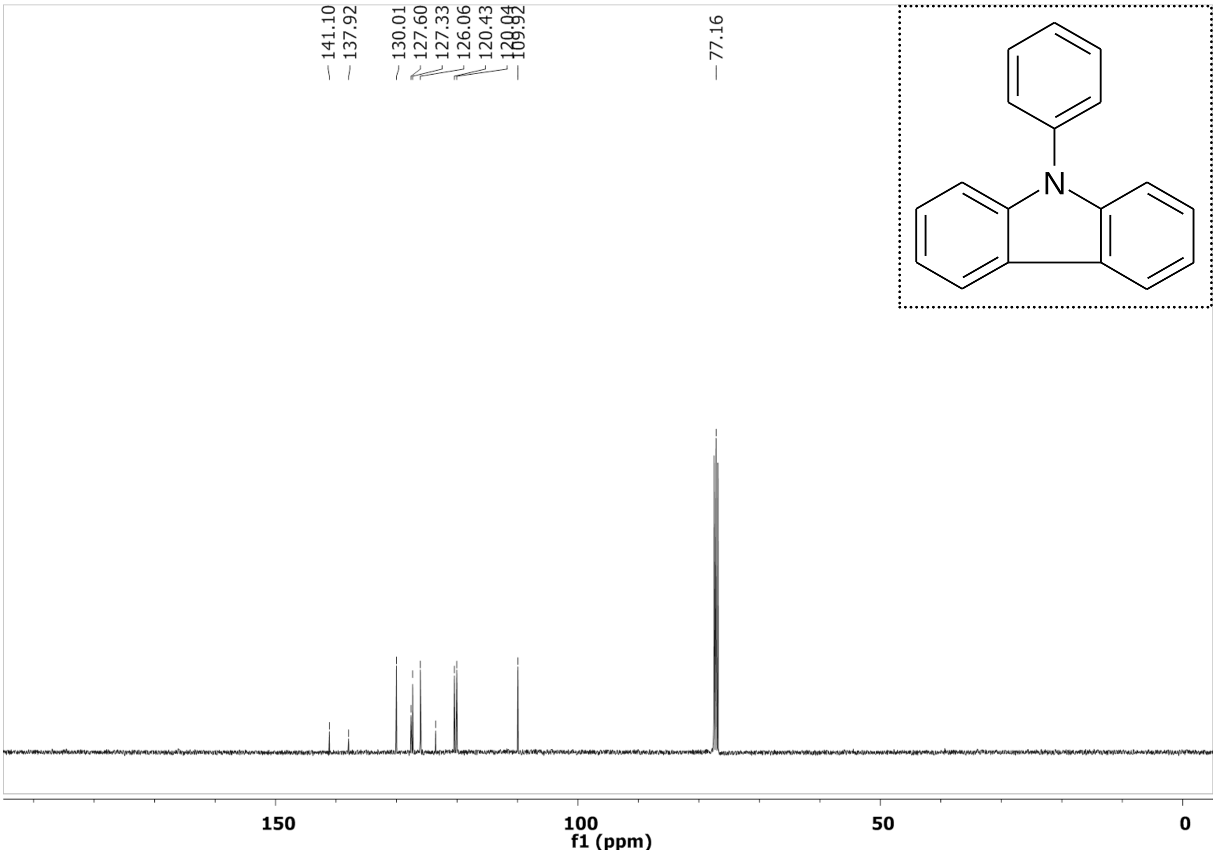

9-(*p*-Tolyl)-9*H*-carbazole (3b)

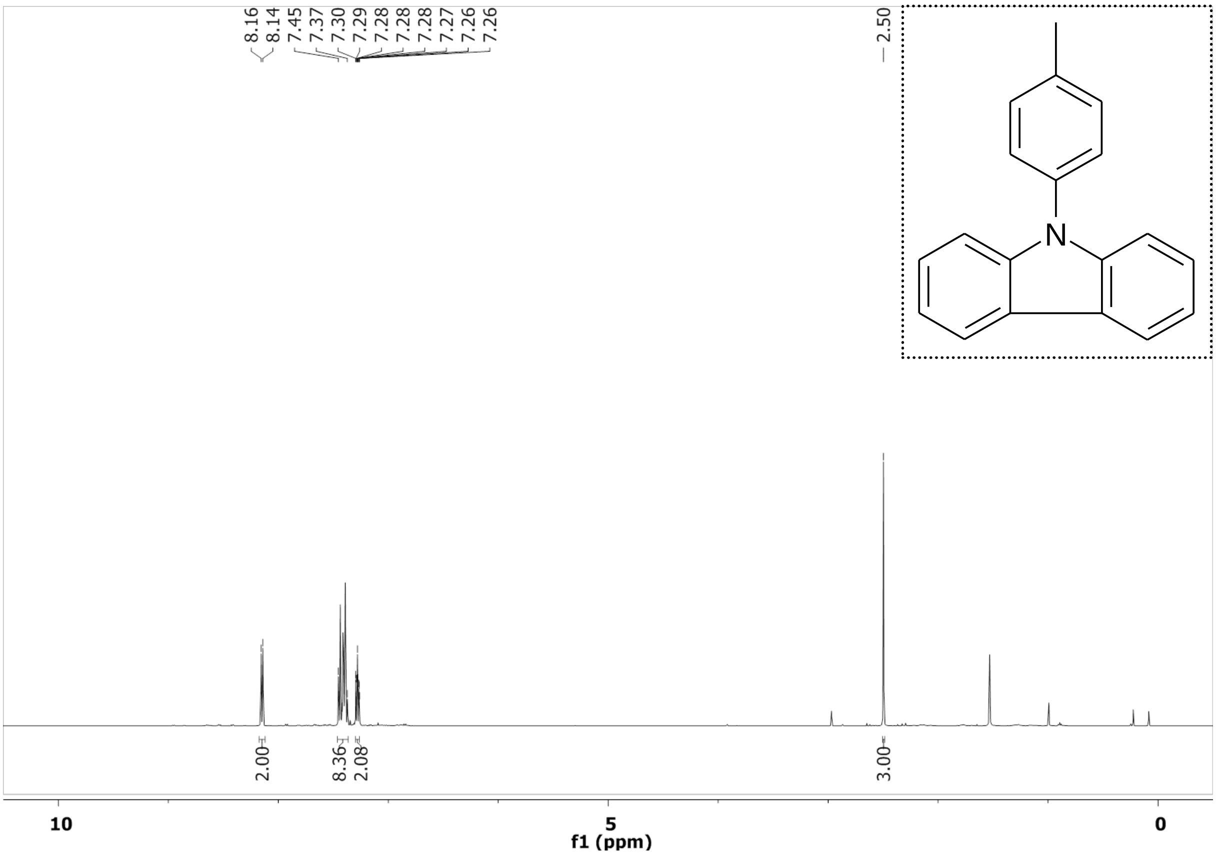

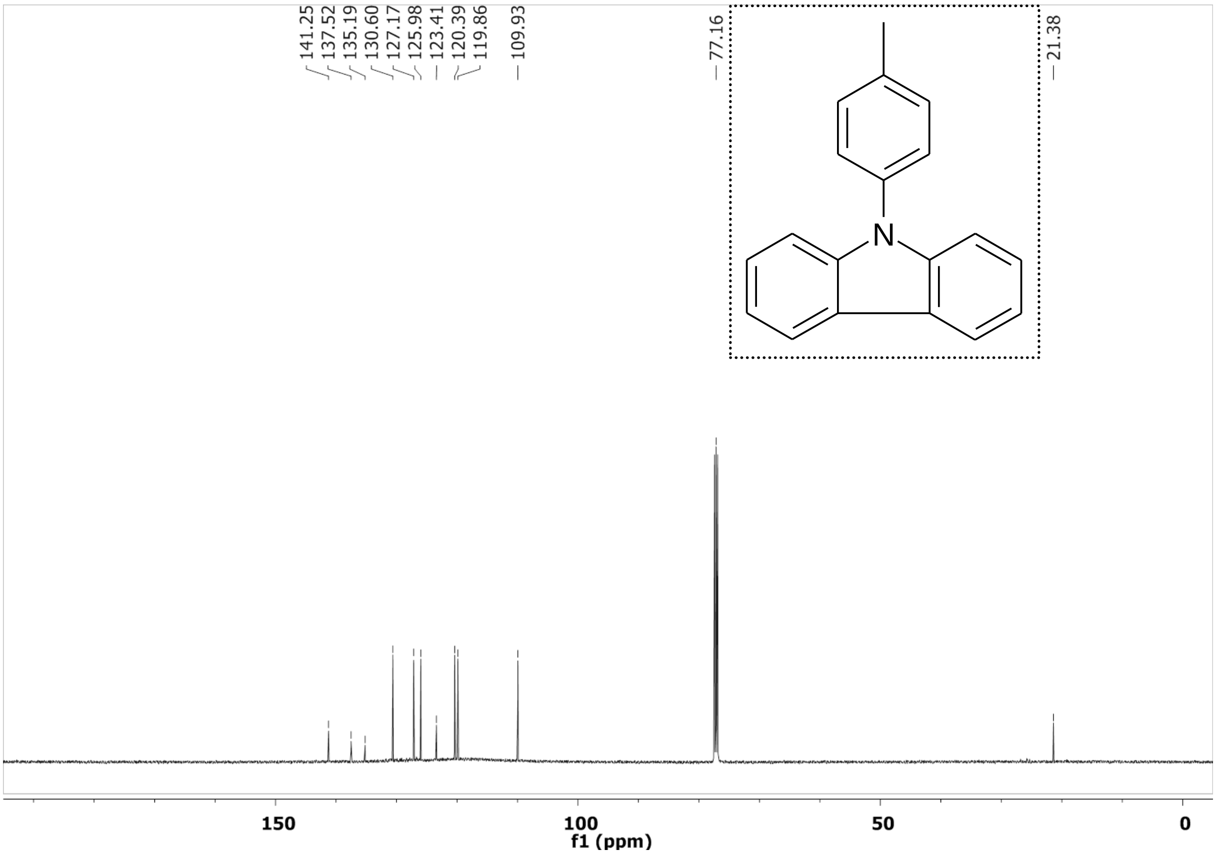

9-Benzyl-9*H*-carbazole (3c)

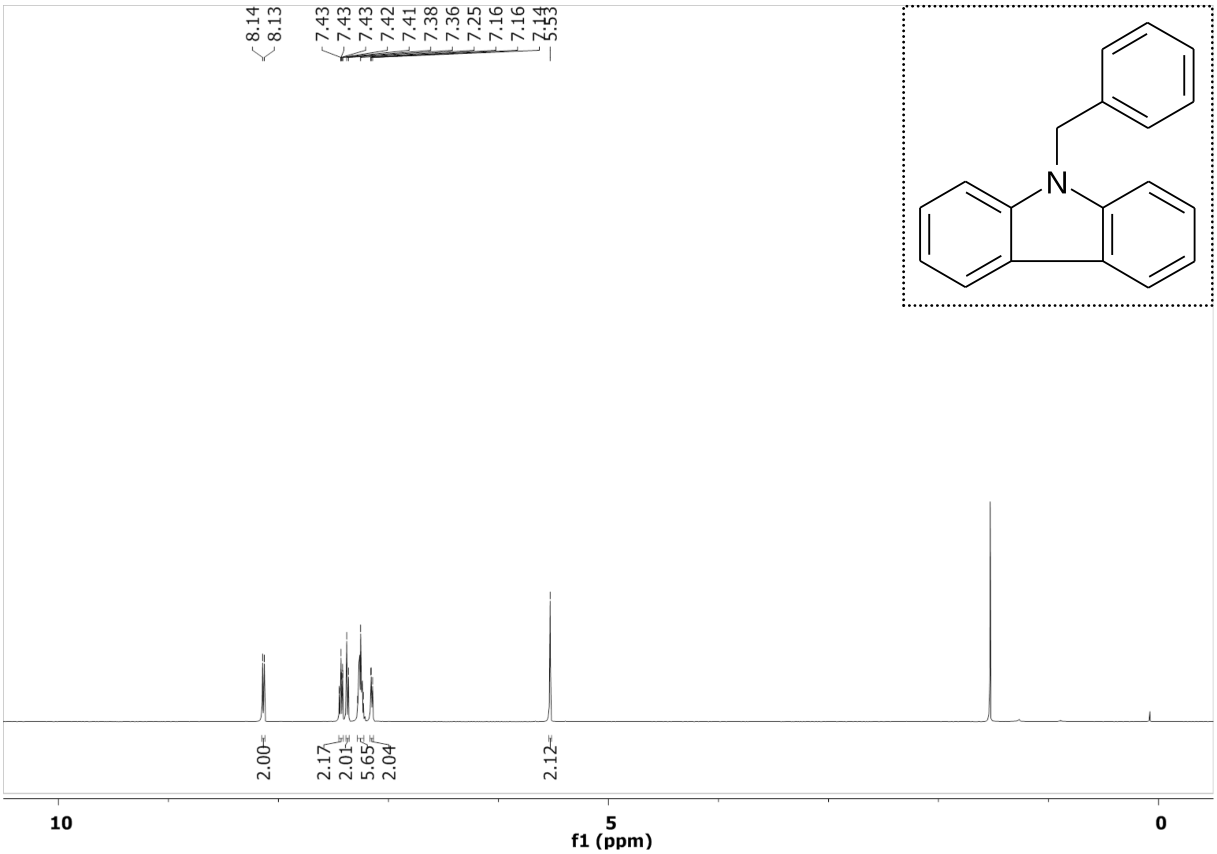

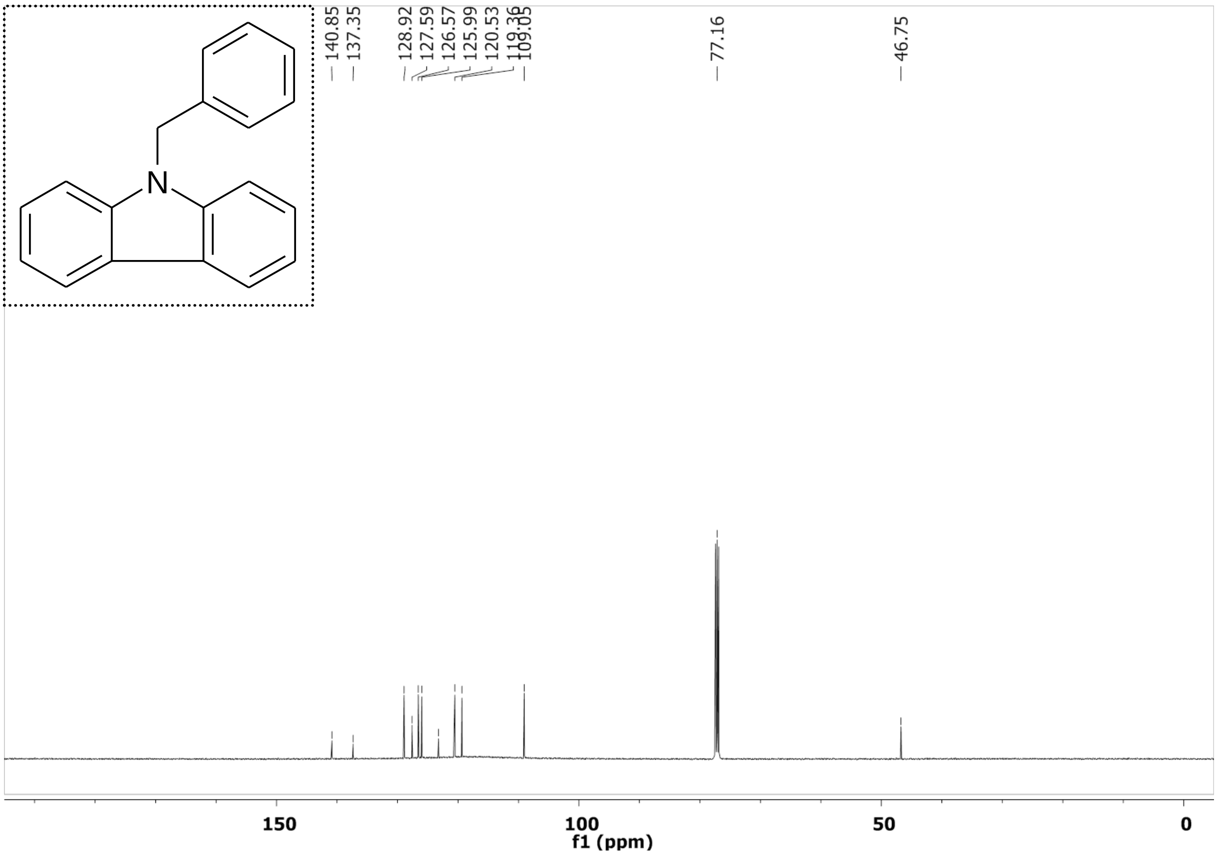

9-(*tert*-Butyl)-9*H*-carbazole (3d)

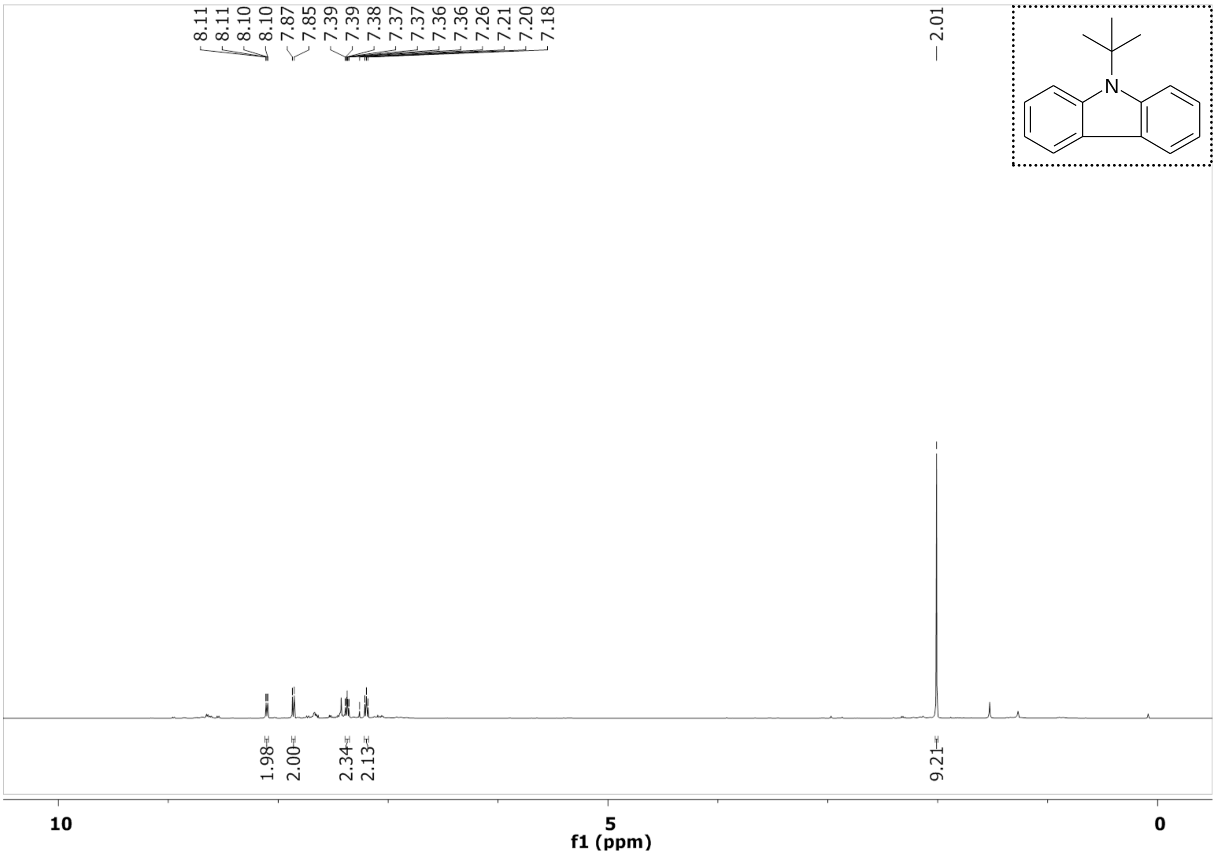

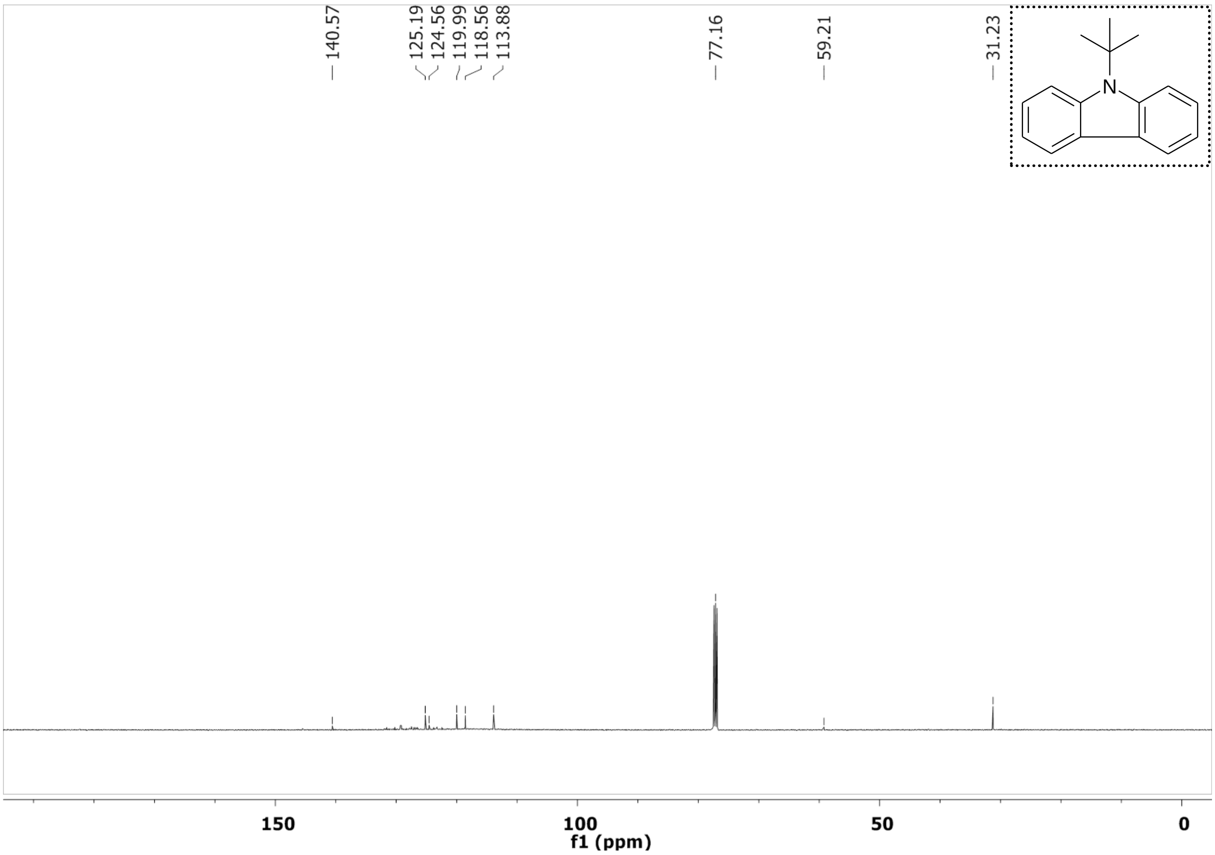

9-Propyl-9*H*-carbazole (3e)

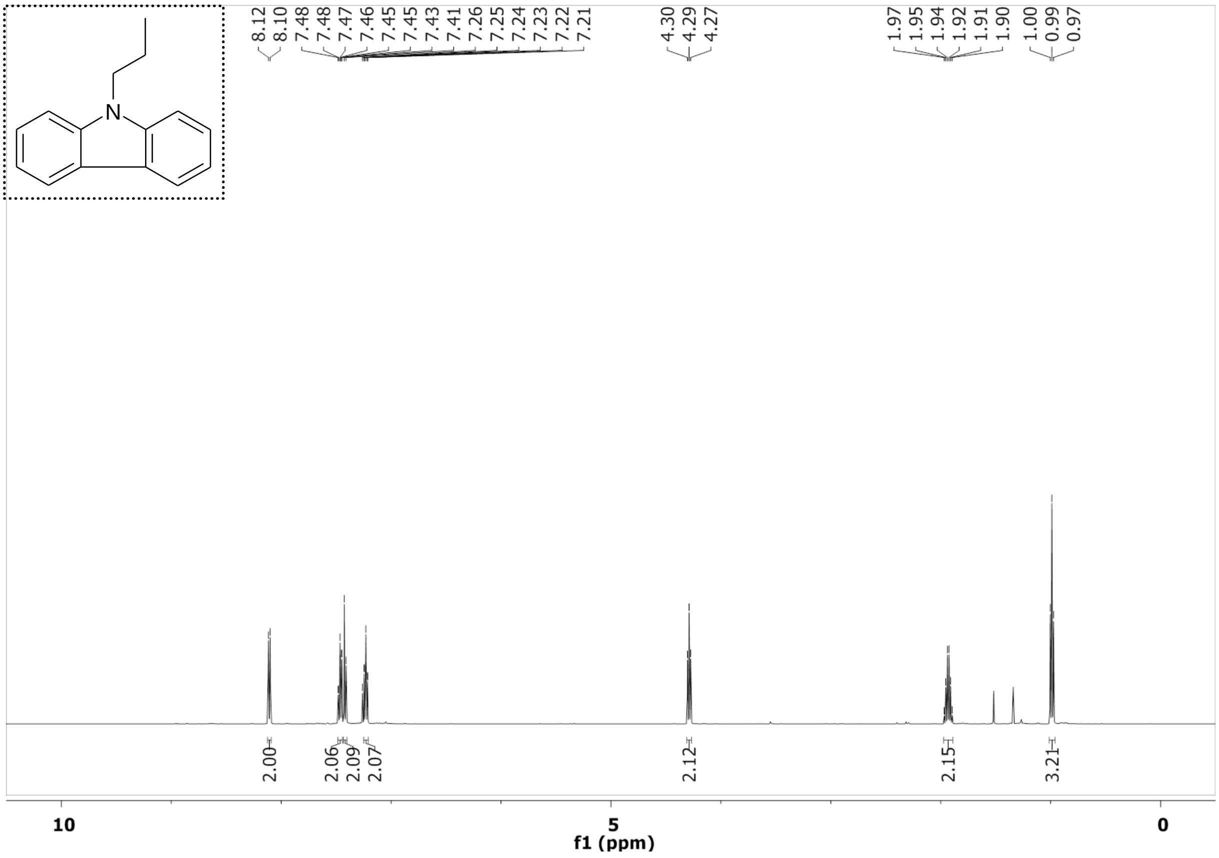

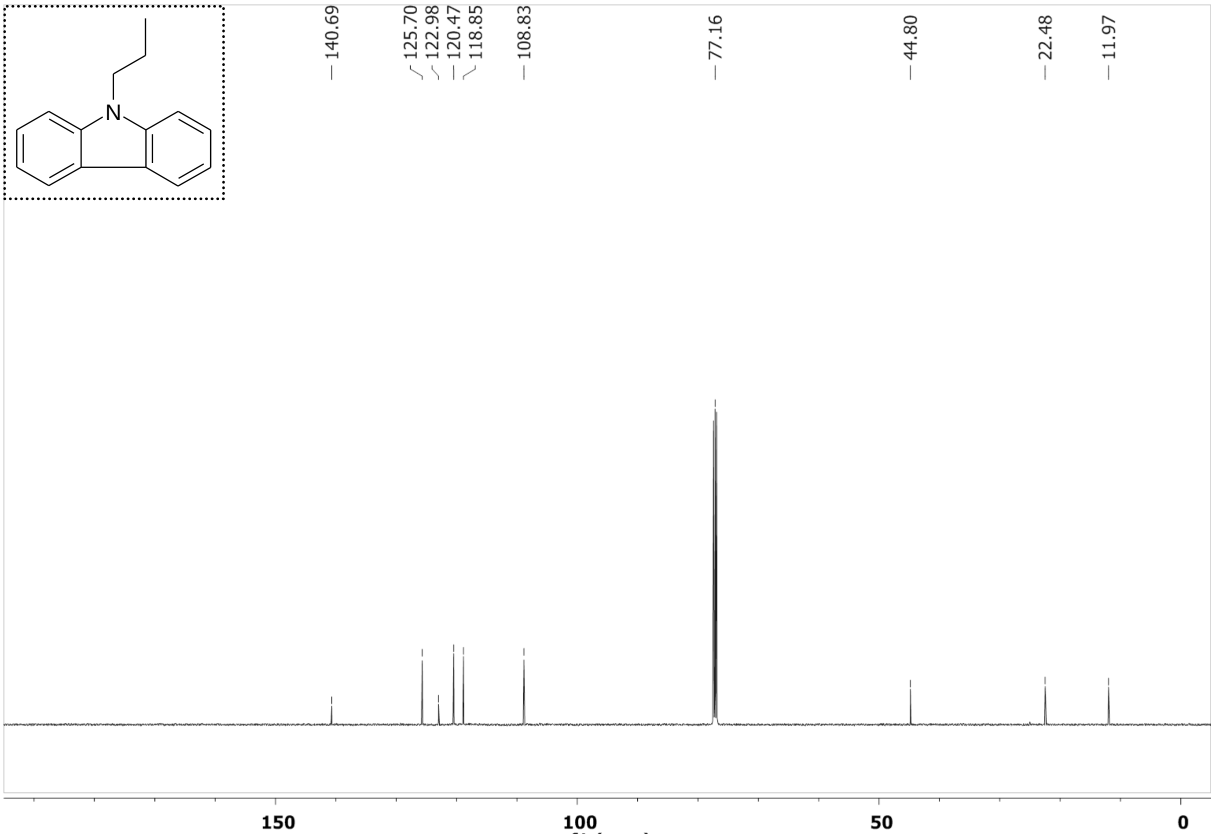

9-(4-Fluorophenyl)-9*H*-carbazole (3f)

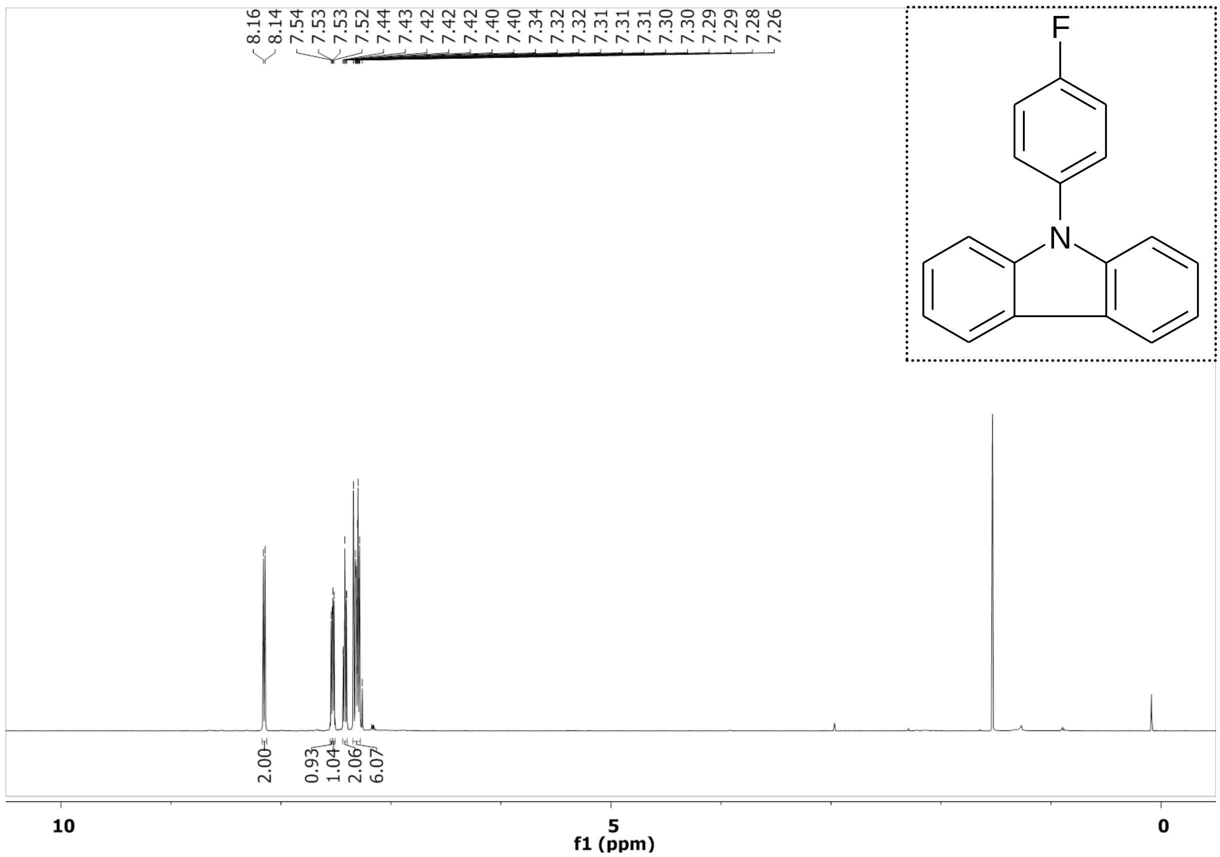

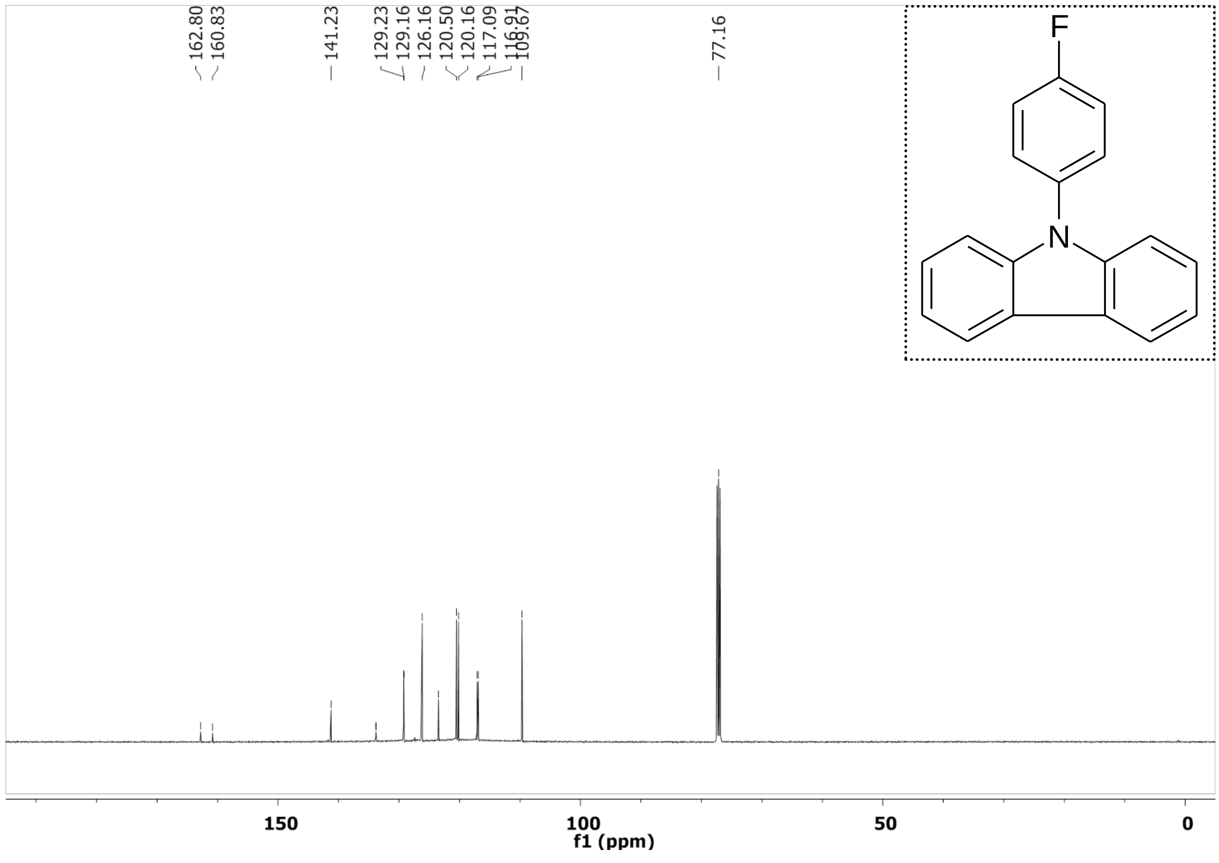

4-(9*H*-Carbazol-9-yl)benzonitrile (3g)

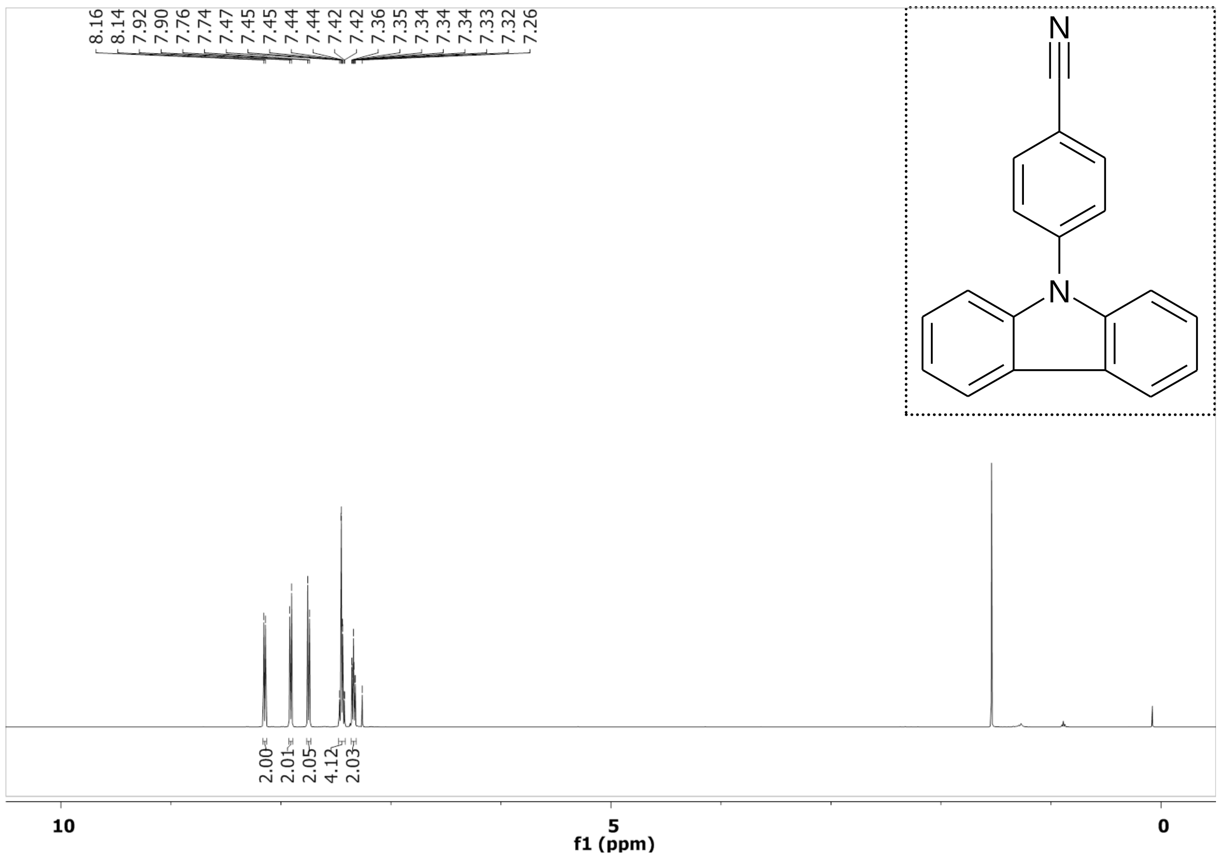

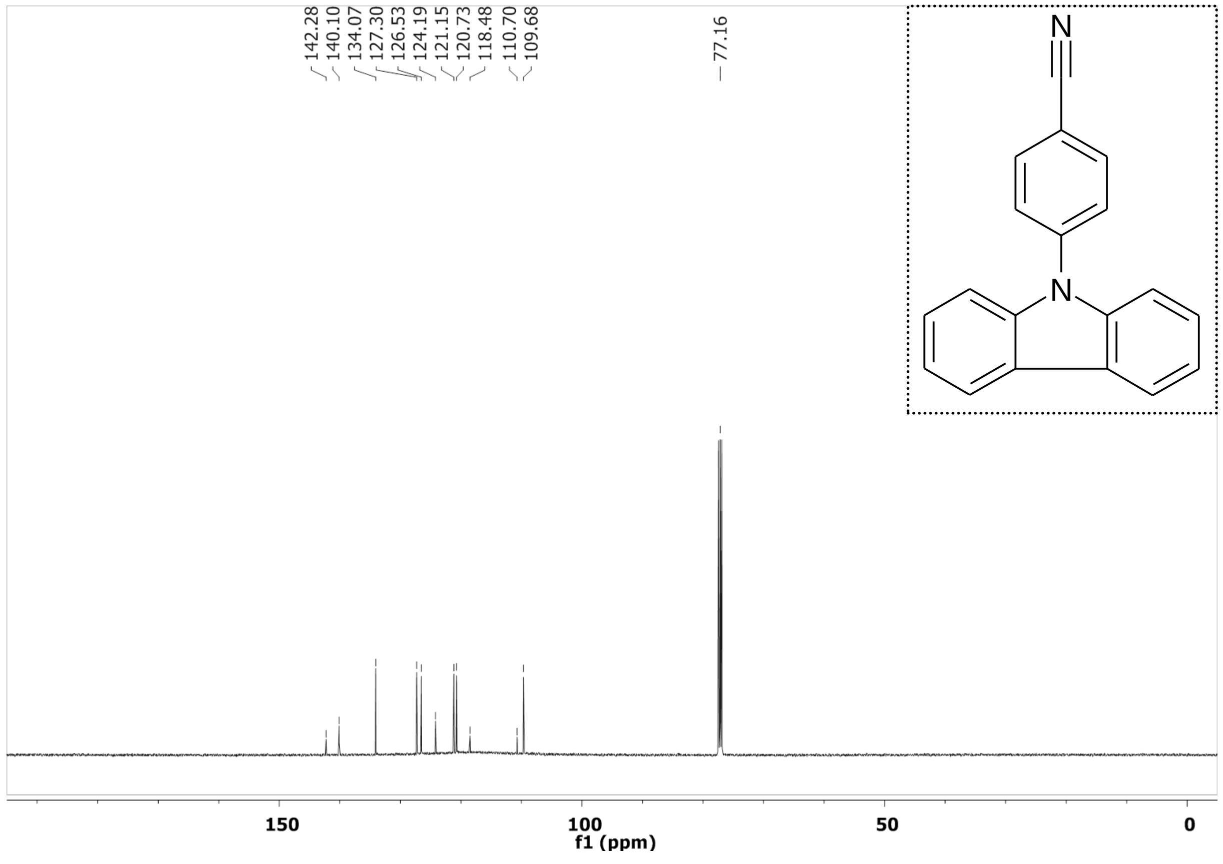

9-(4-Chlorophenyl)-9*H*-carbazole (3h)

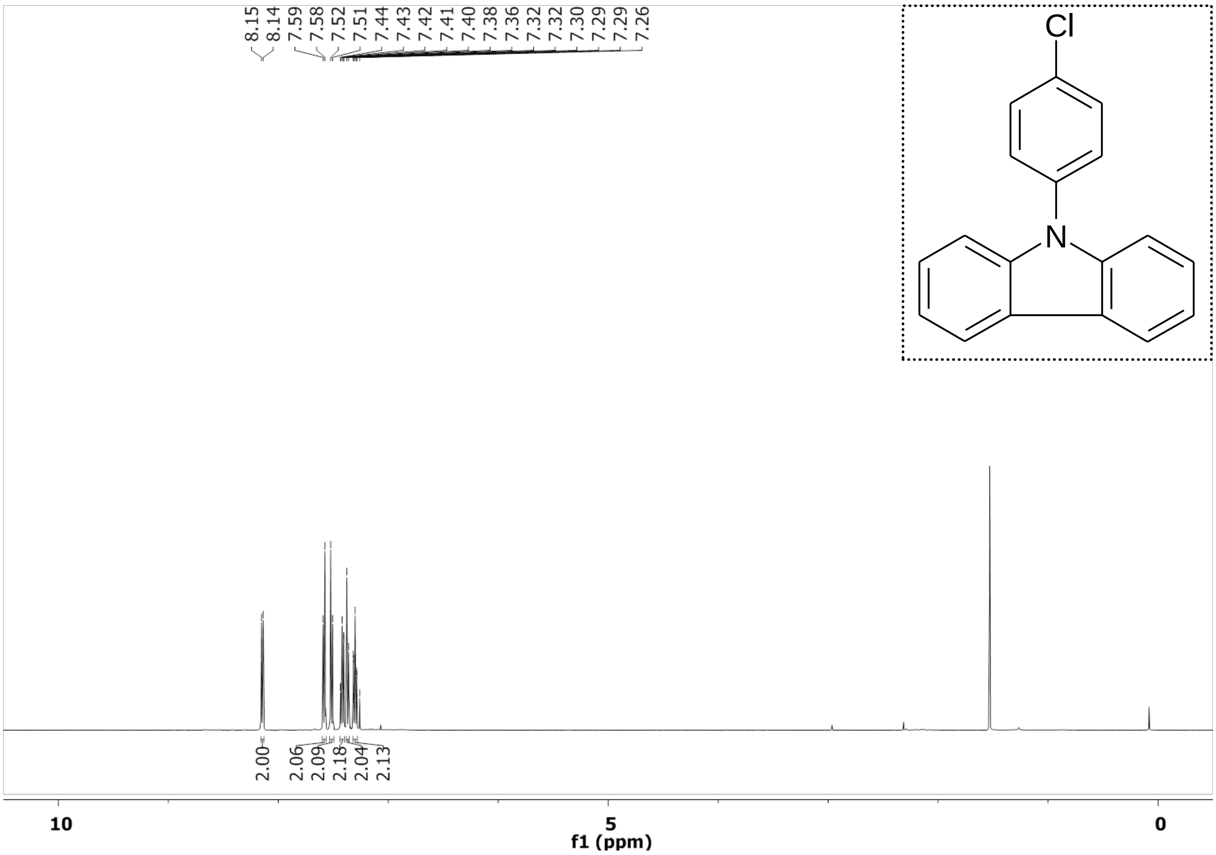

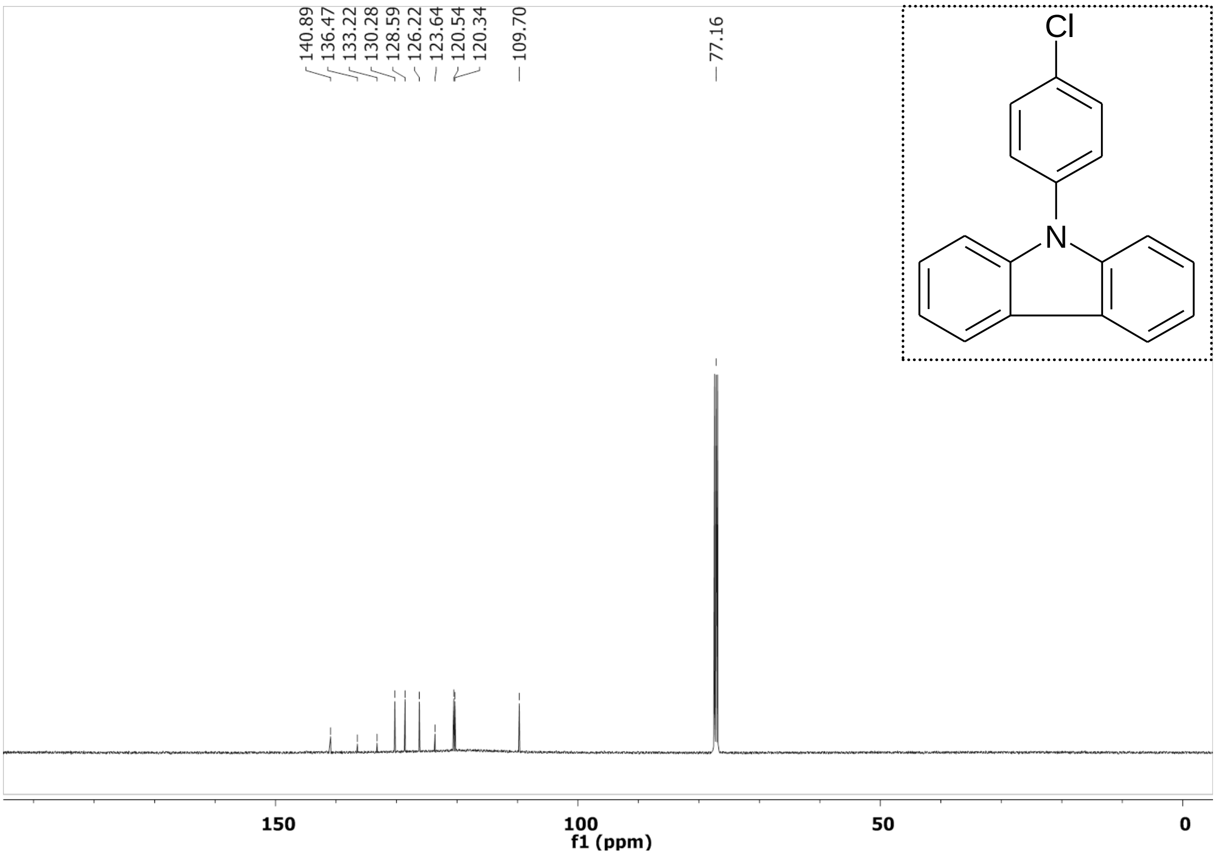

9-(4-(Trifluoromethyl)phenyl)-9*H*-carbazole (3i)

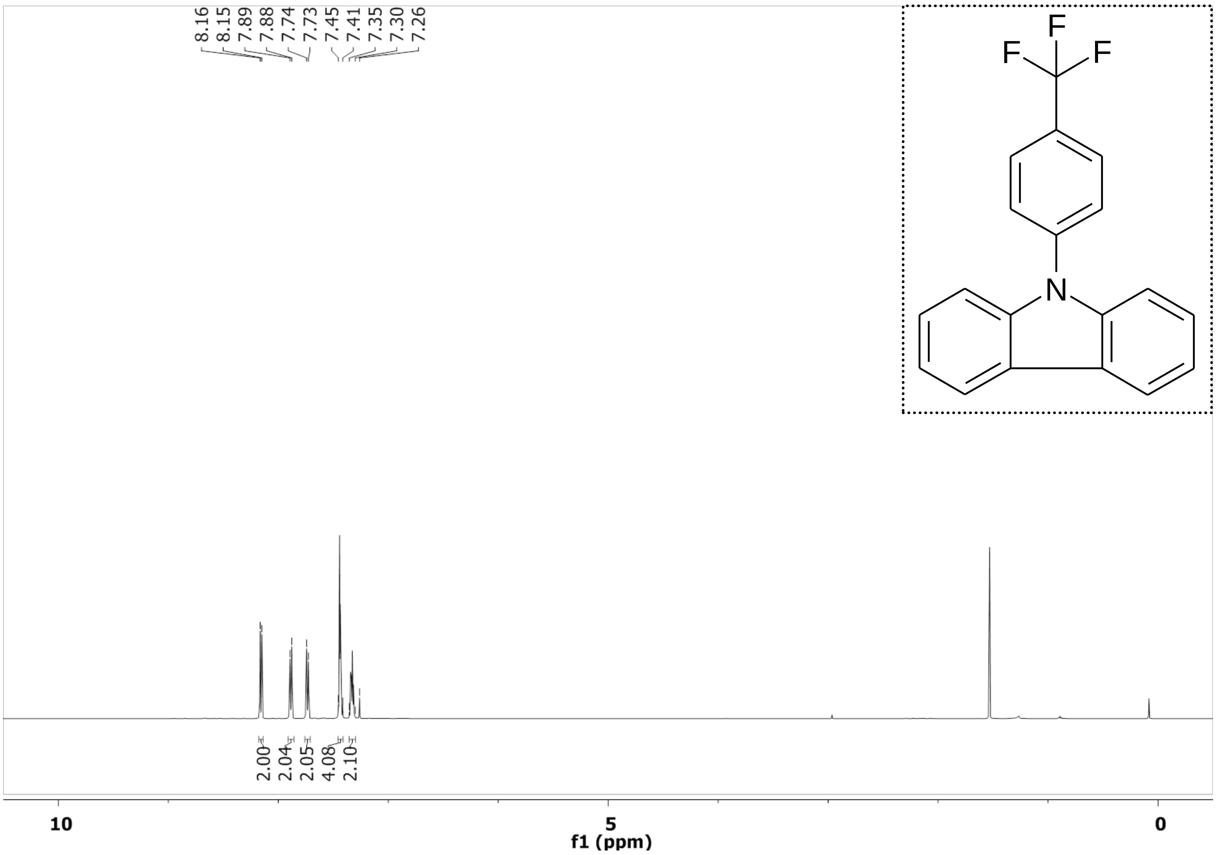

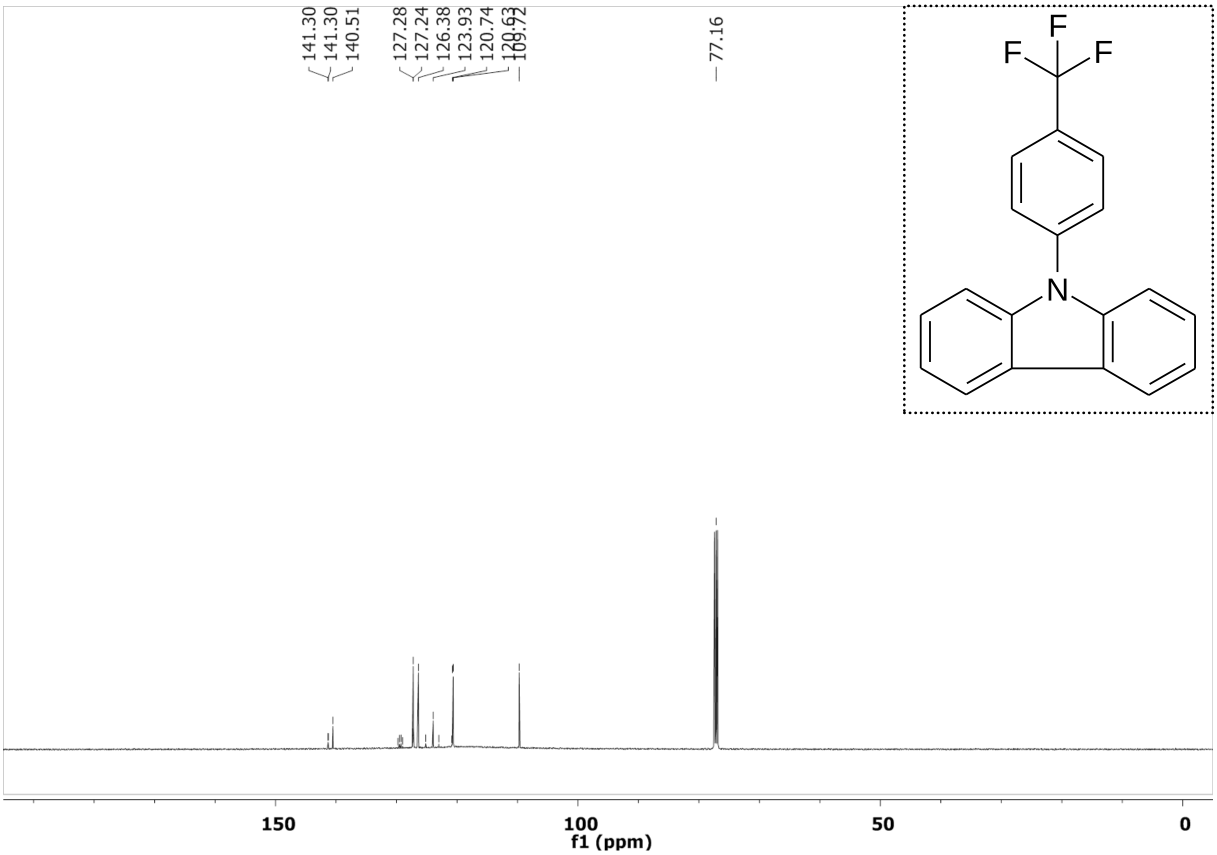

9-(3,5-Difluorophenyl)-9*H*-carbazole (3j)

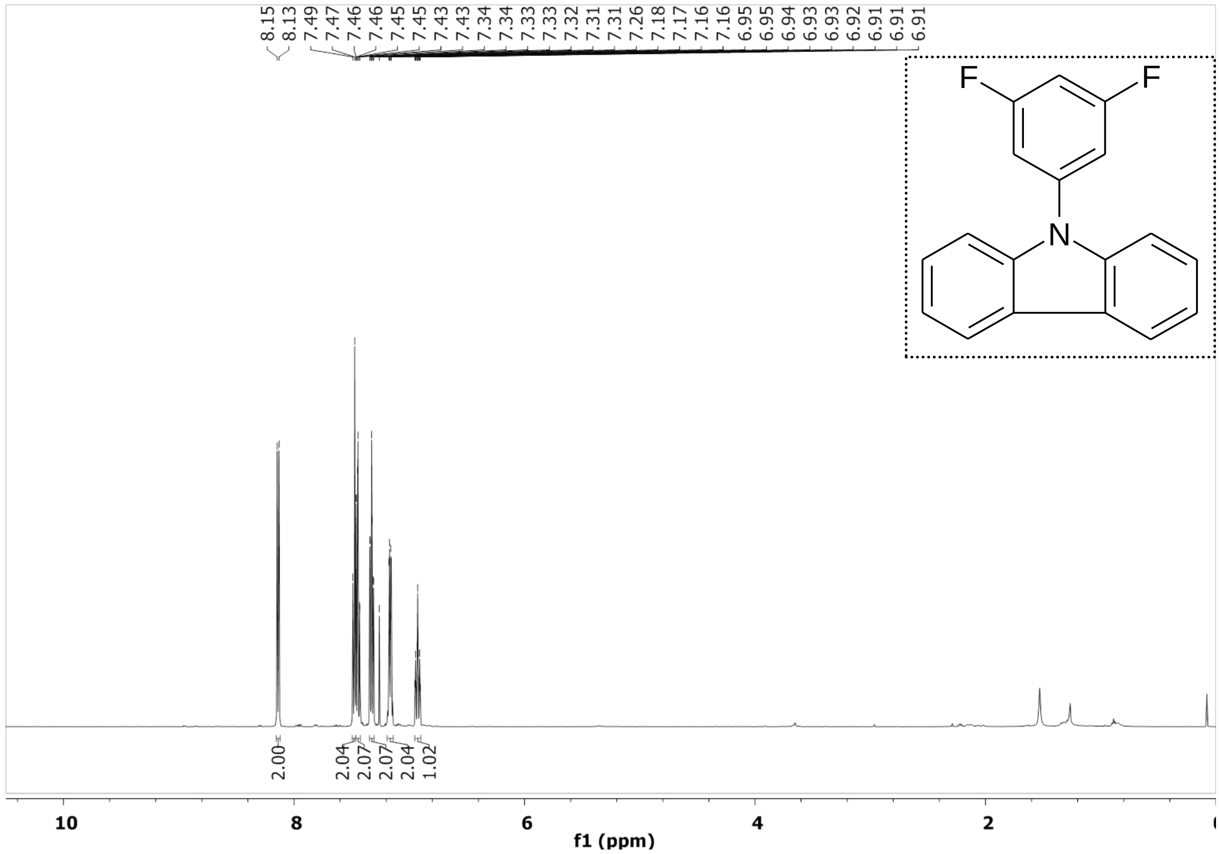

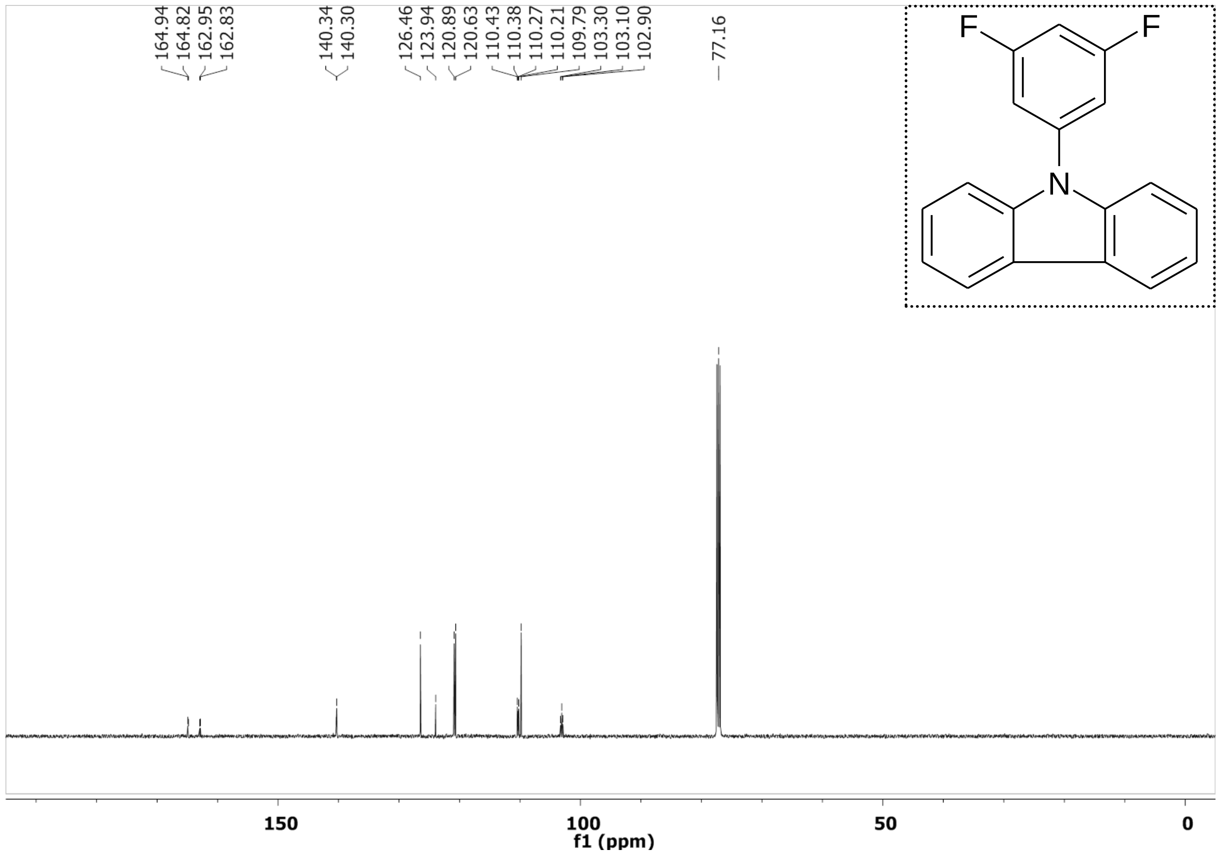

Methyl 4-(9*H*-carbazol-9-yl)benzoate (3k)

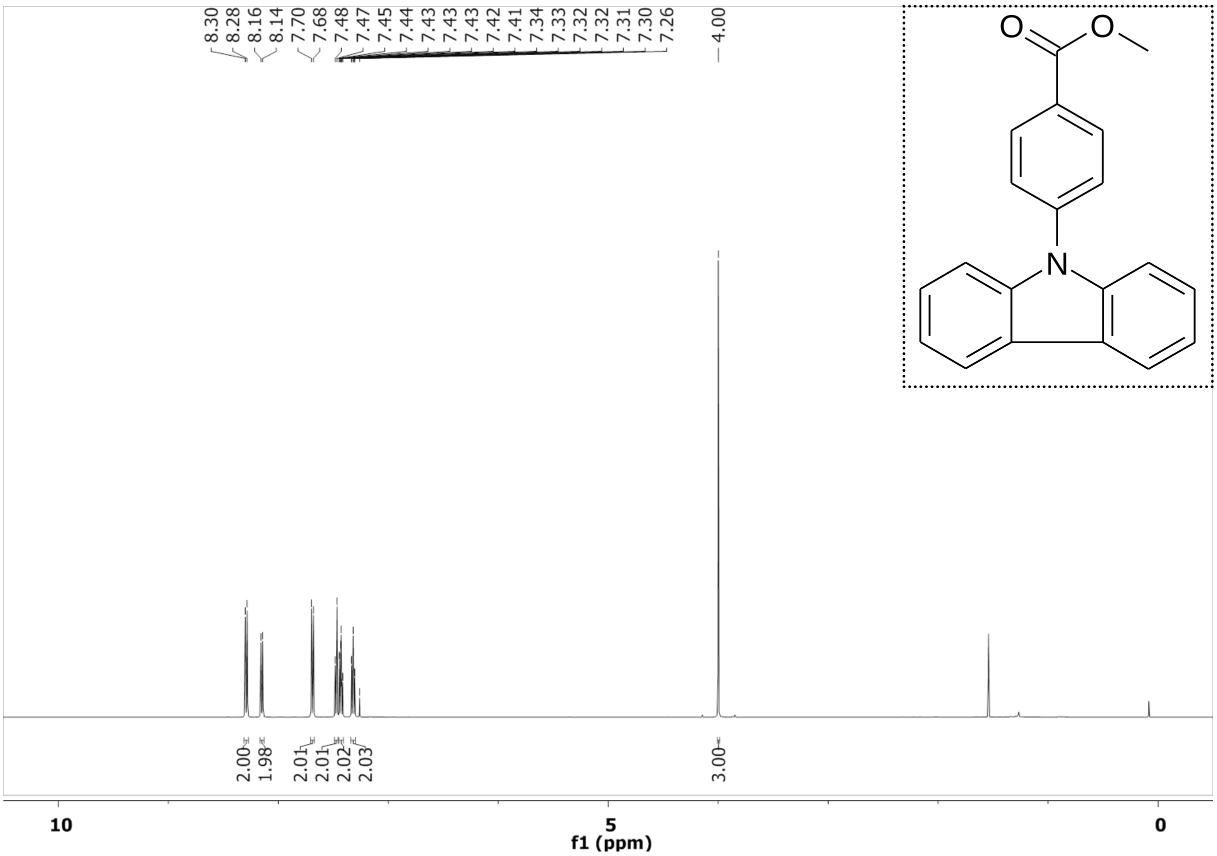

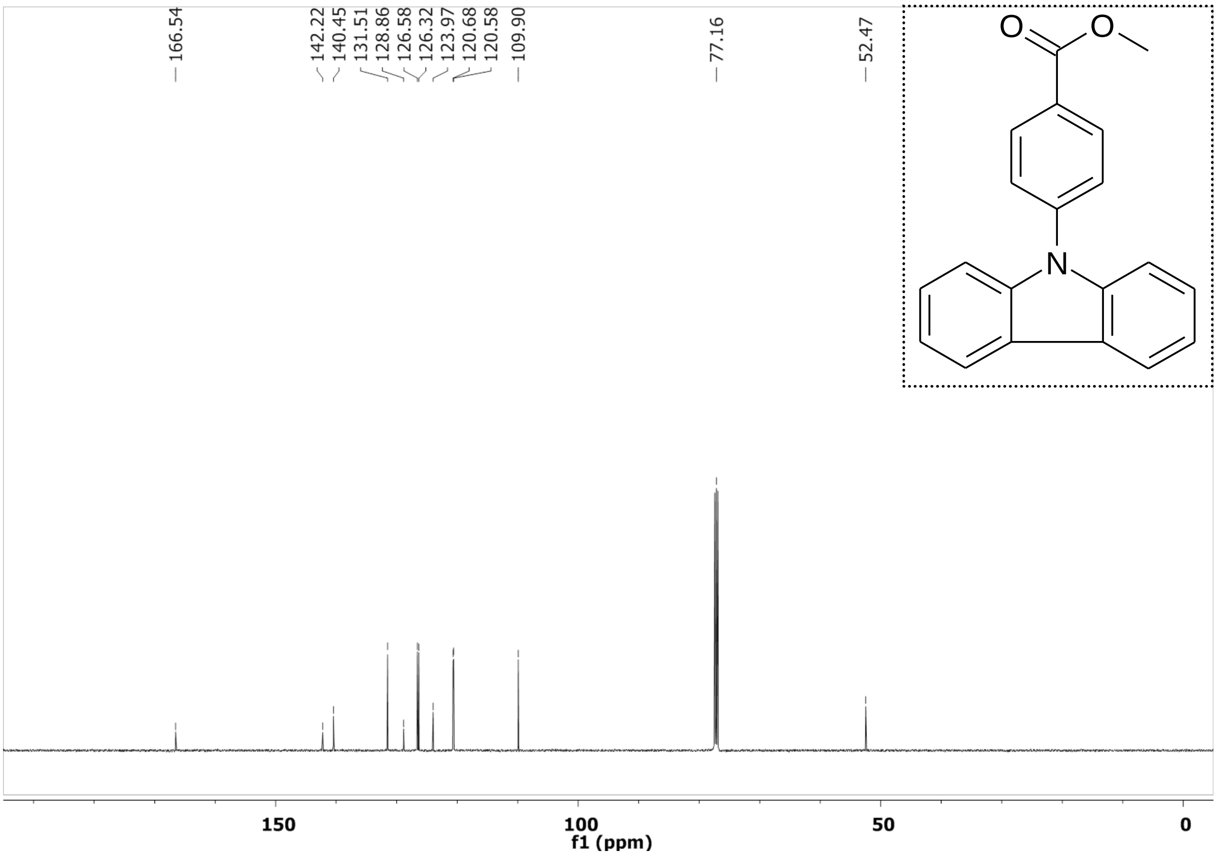

Ethyl 3-(9*H*-carbazol-9-yl)benzoate (3l)

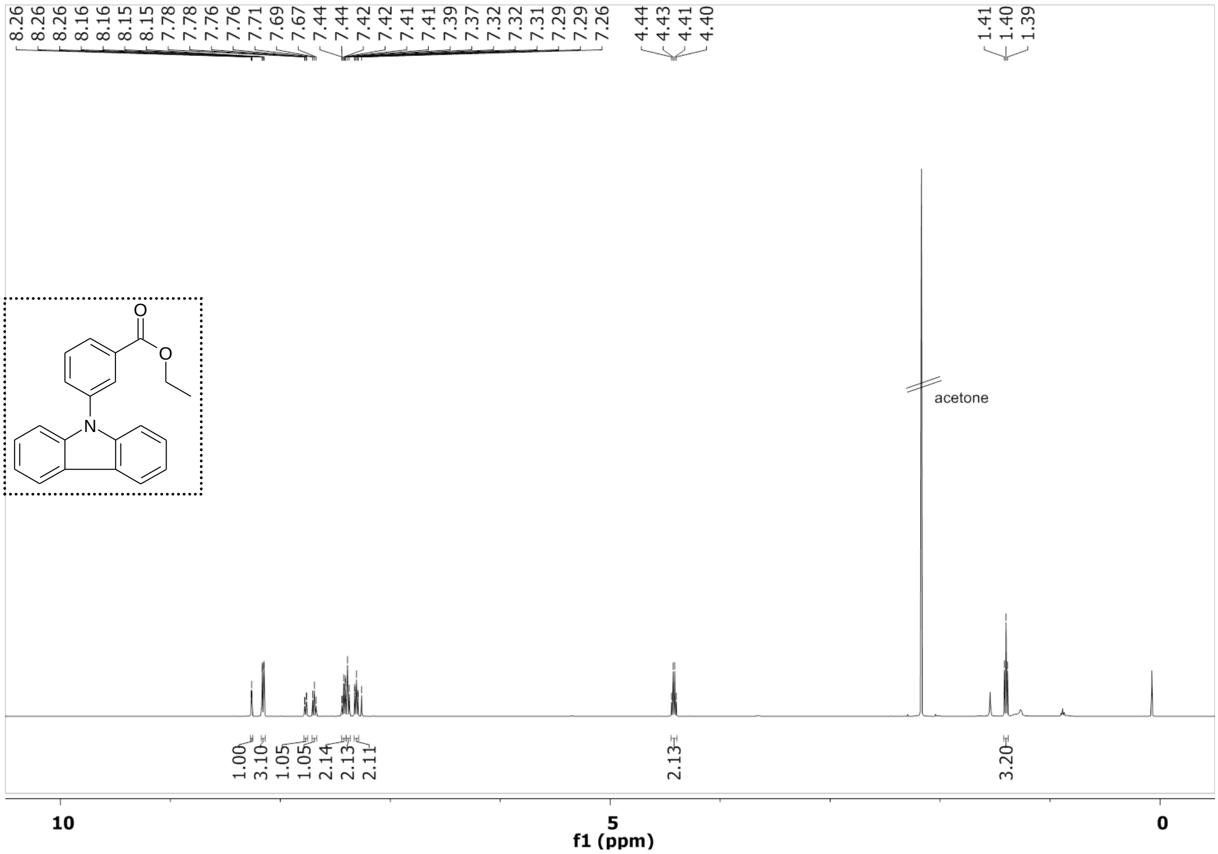

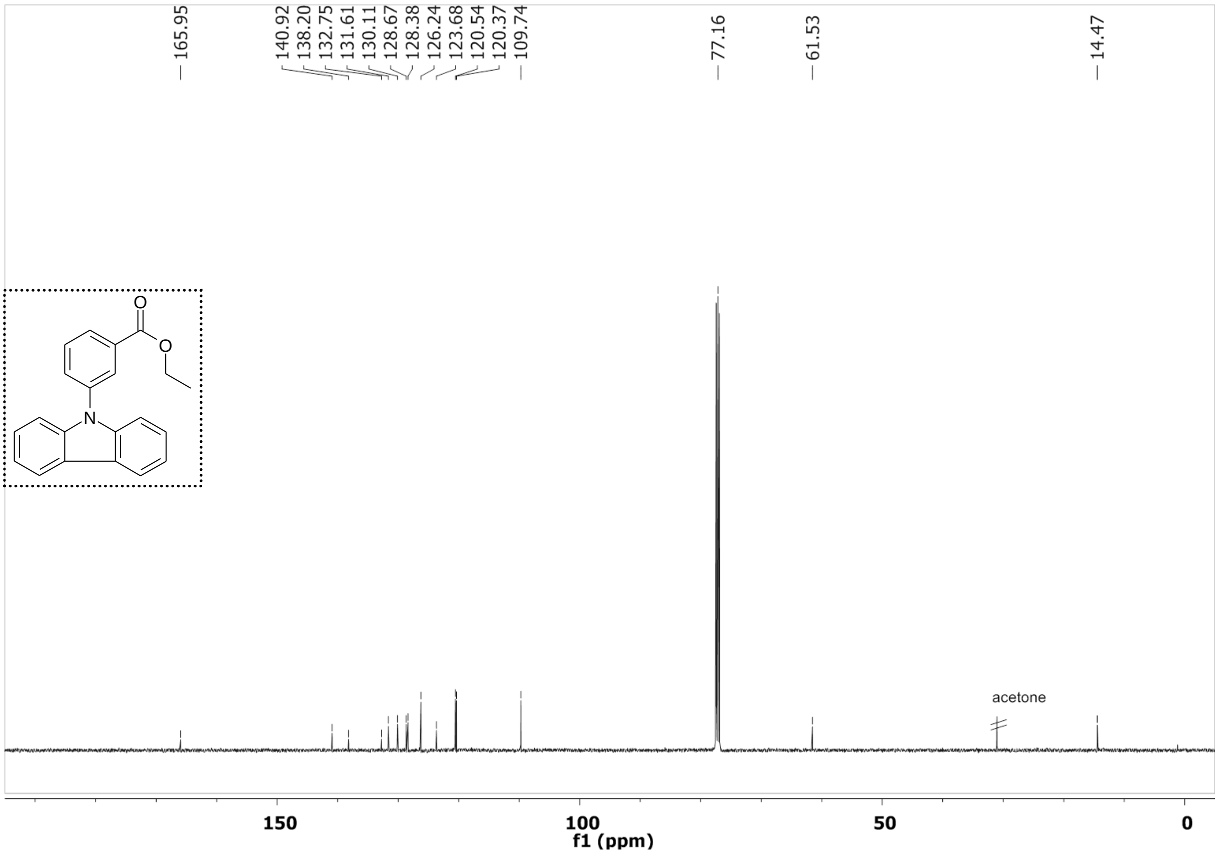

9-(3-Nitrophenyl)-9*H*-carbazole (3m):

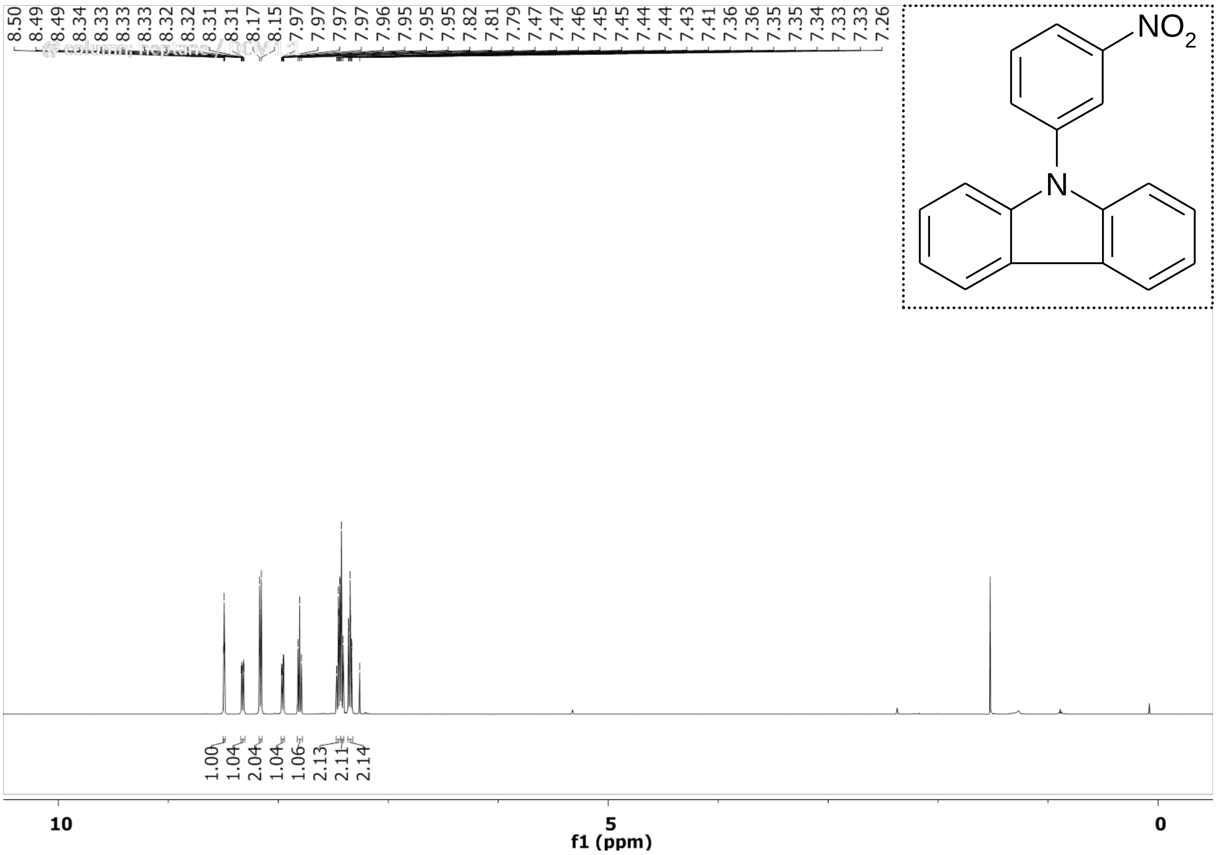

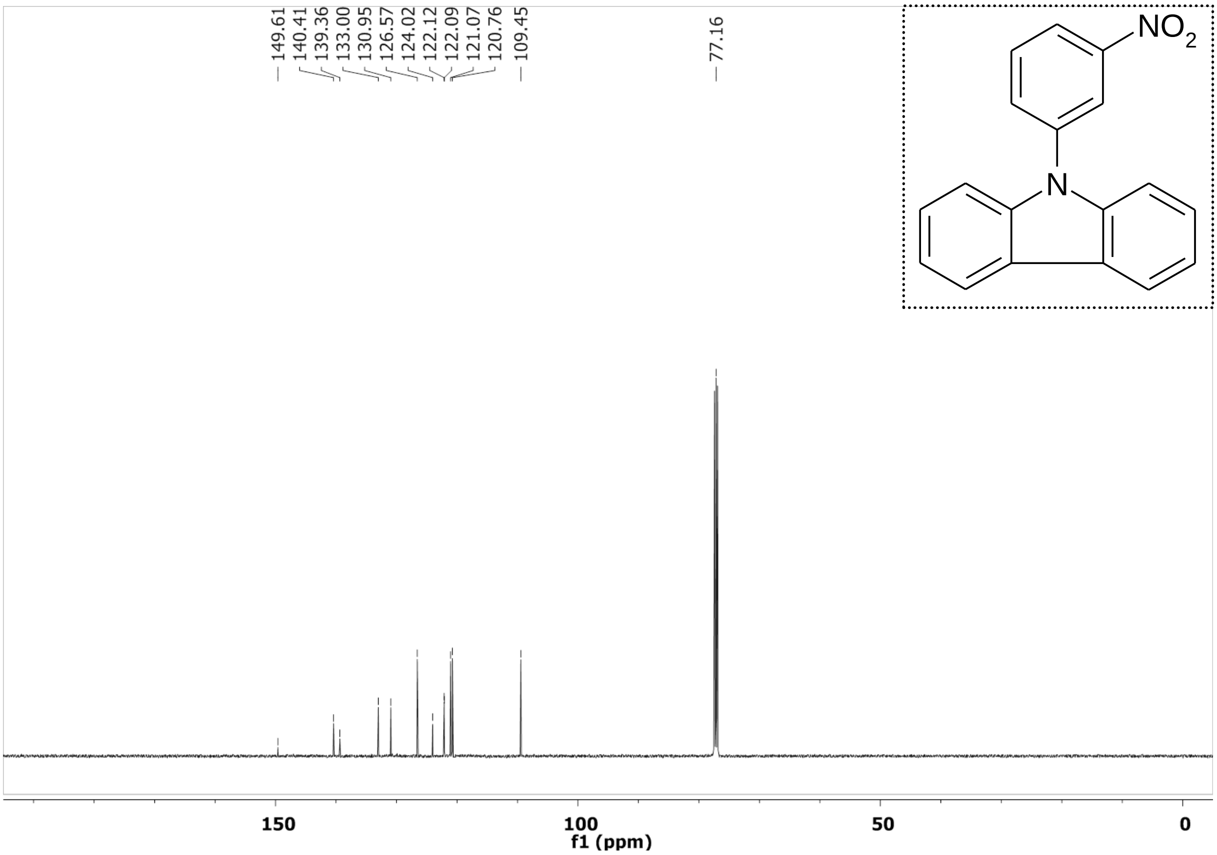

9-([1,1'-Biphenyl]-4-yl)-9*H*-carbazole (3n)

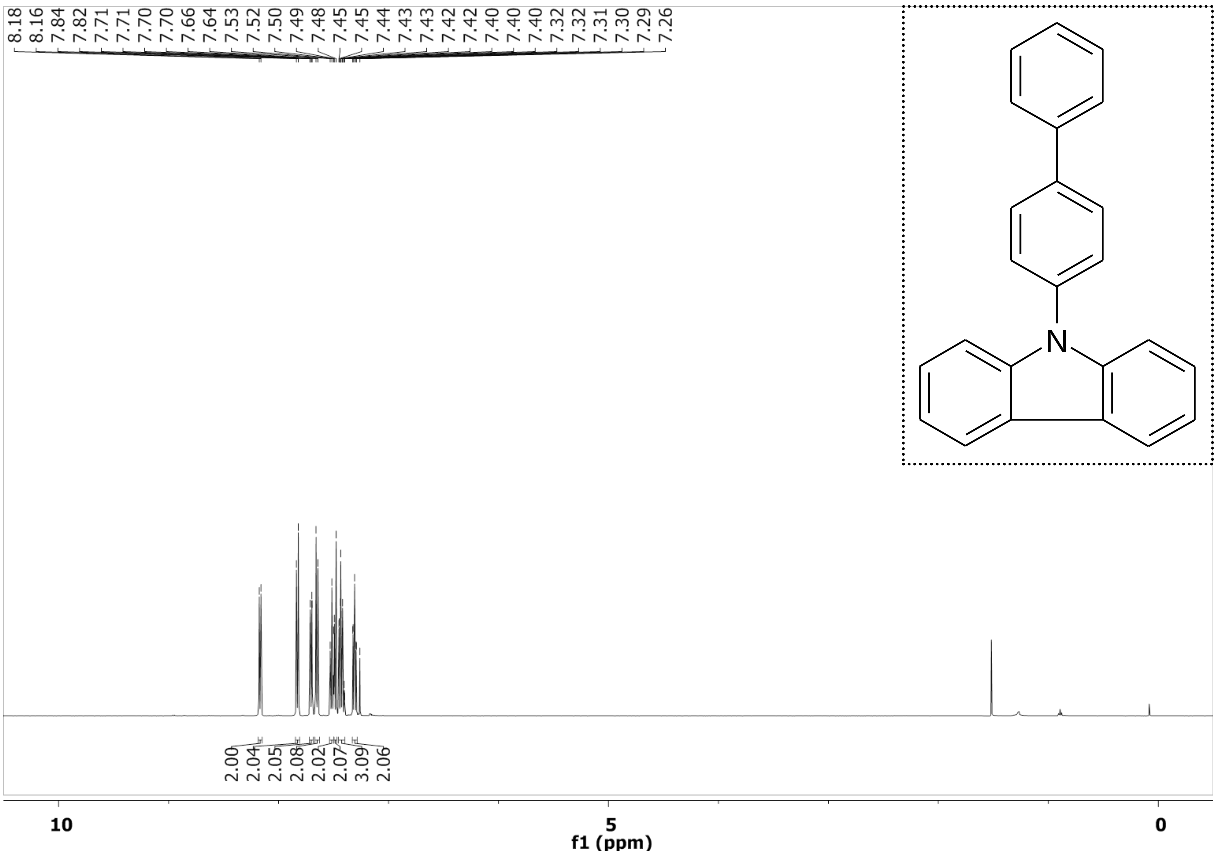

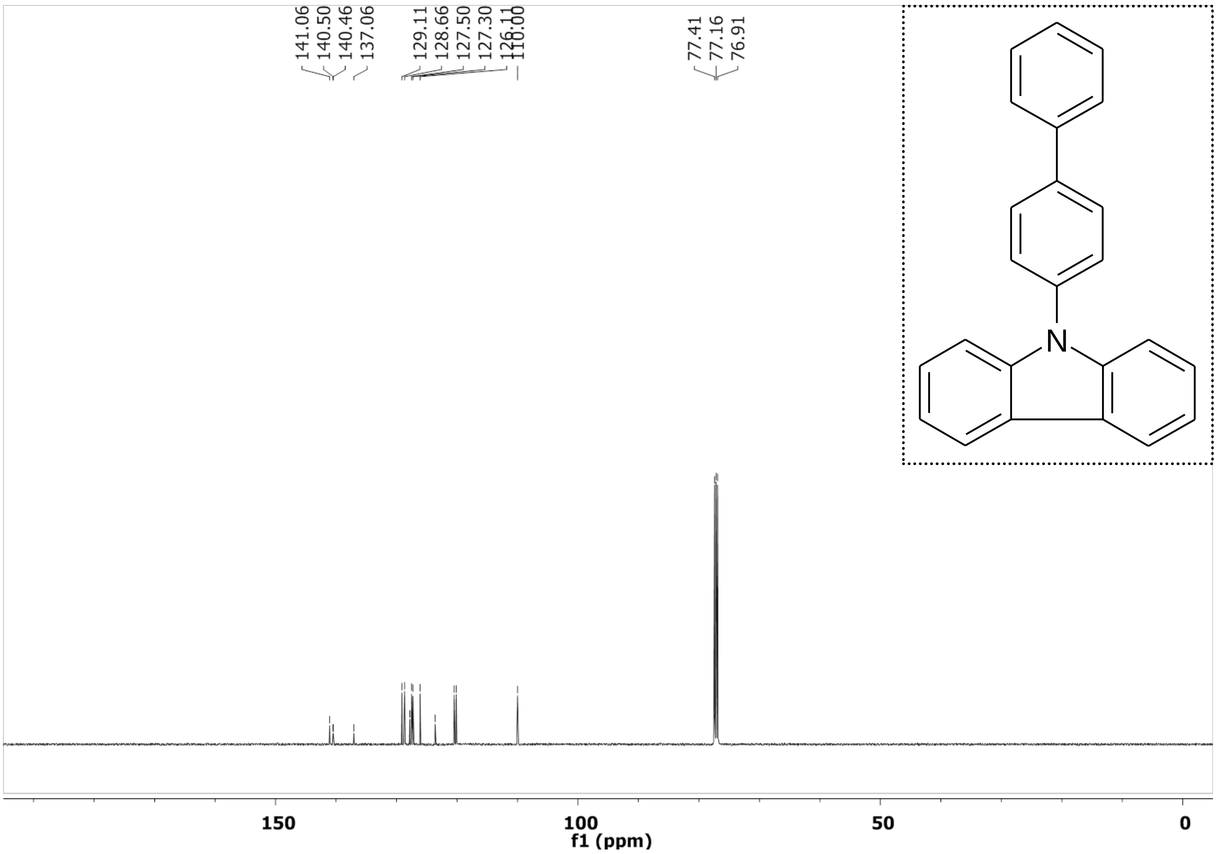

9-(Naphthalen-1-yl)-9*H*-carbazole (3o)

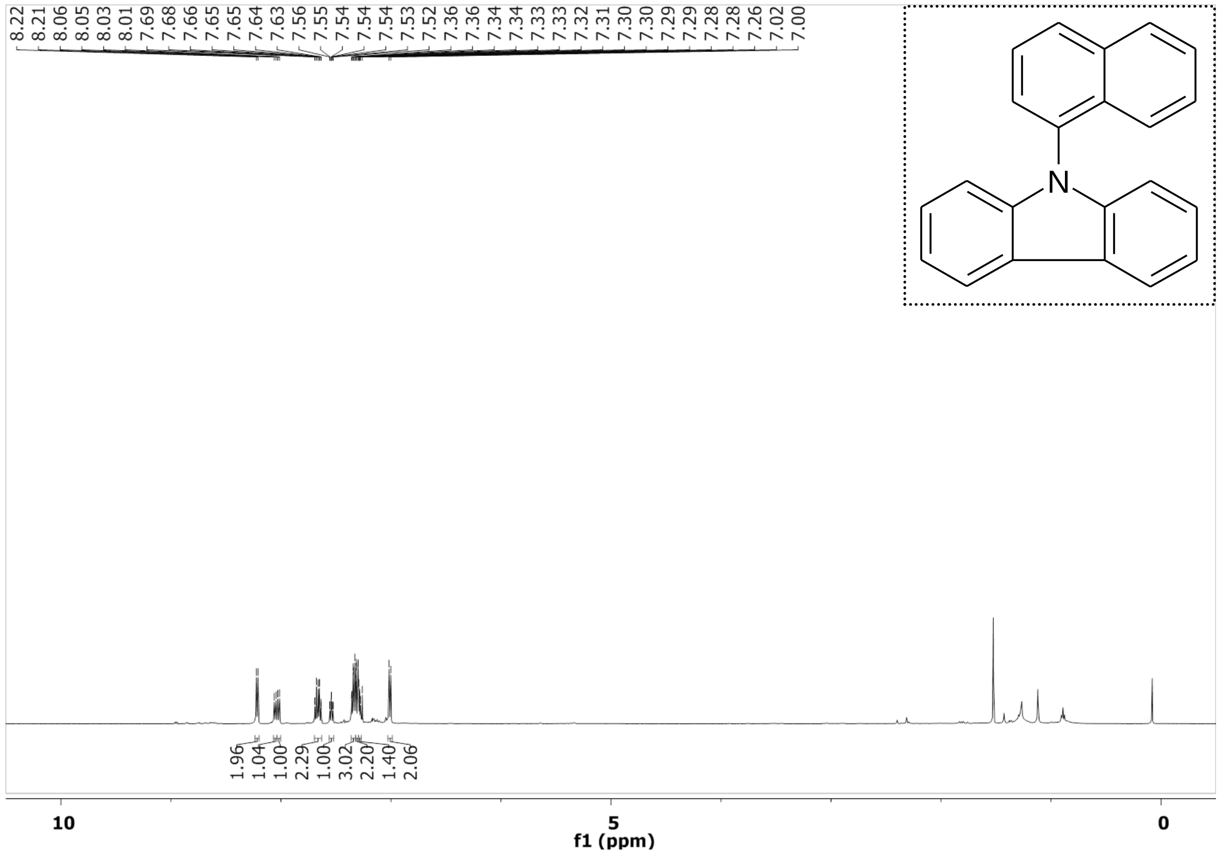

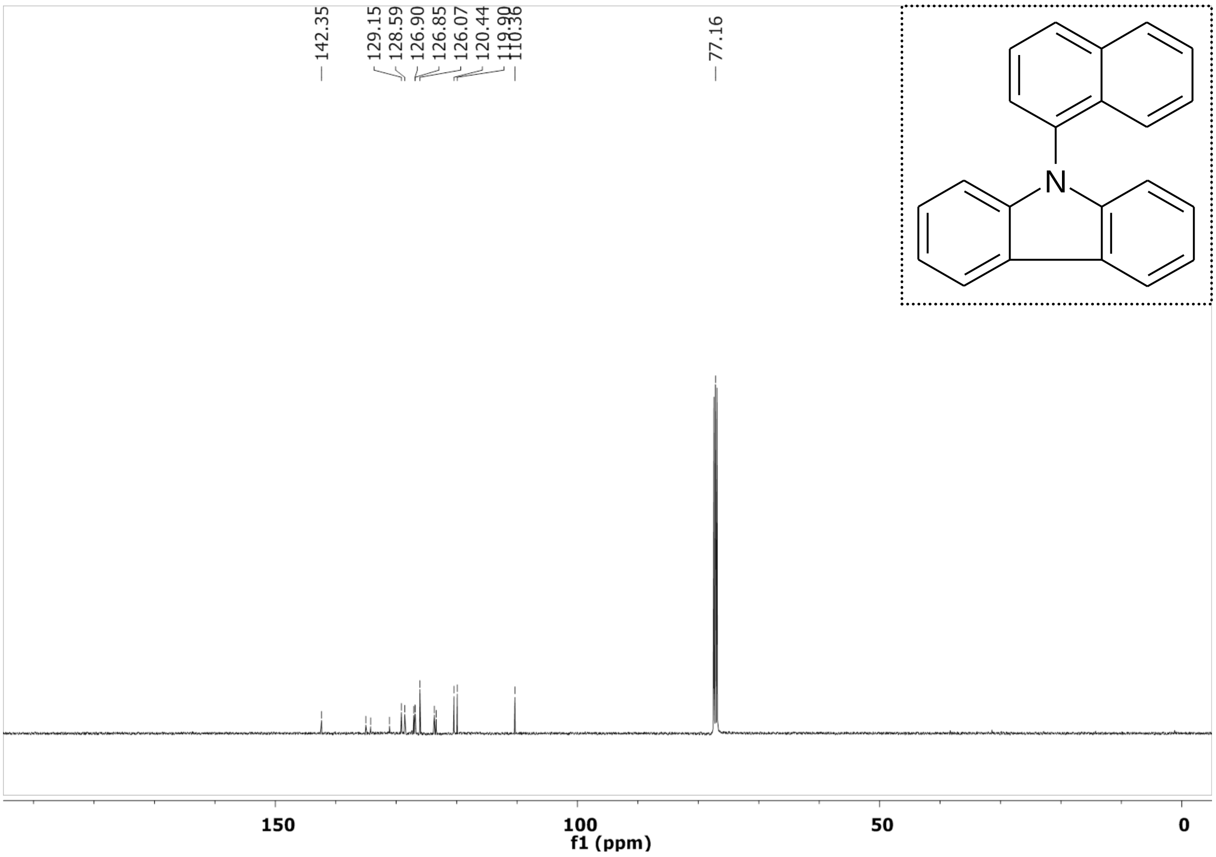

9-(Perfluorophenyl)-9*H*-carbazole (3p)

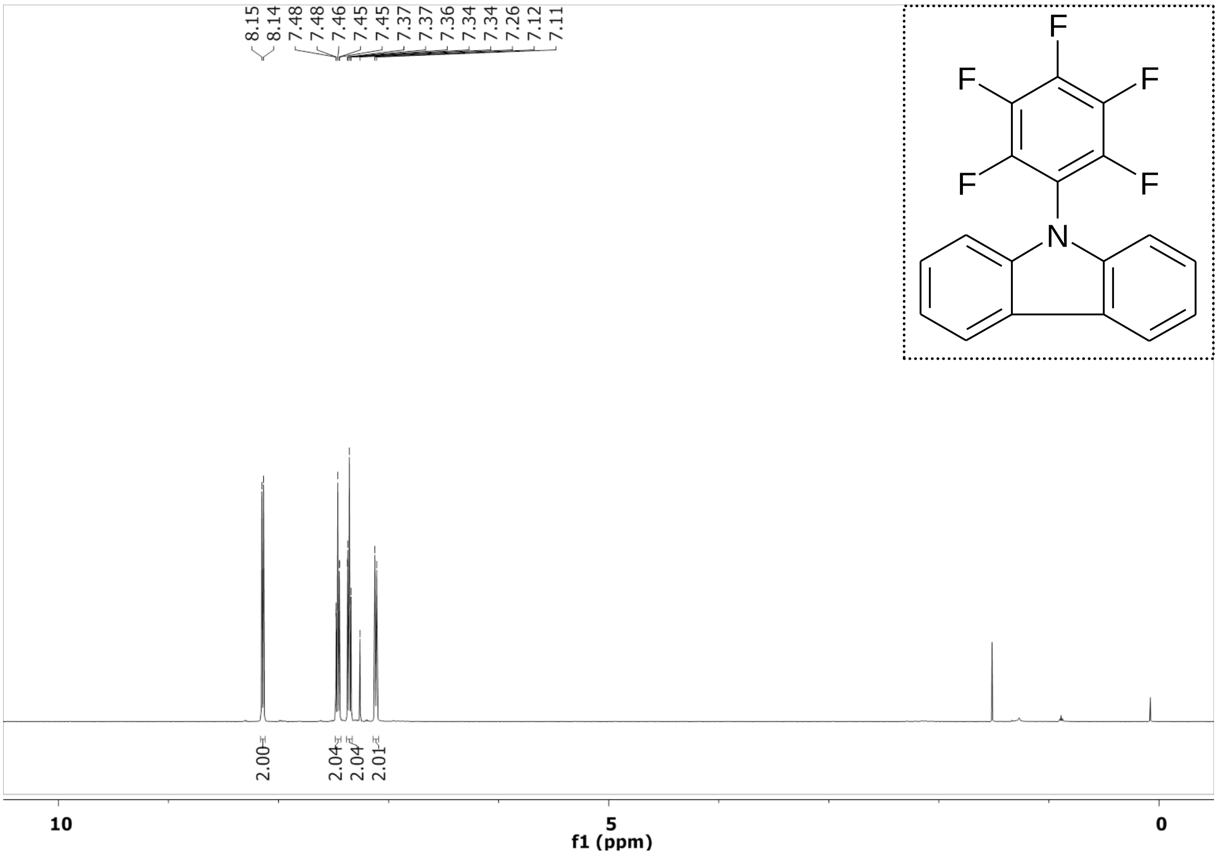

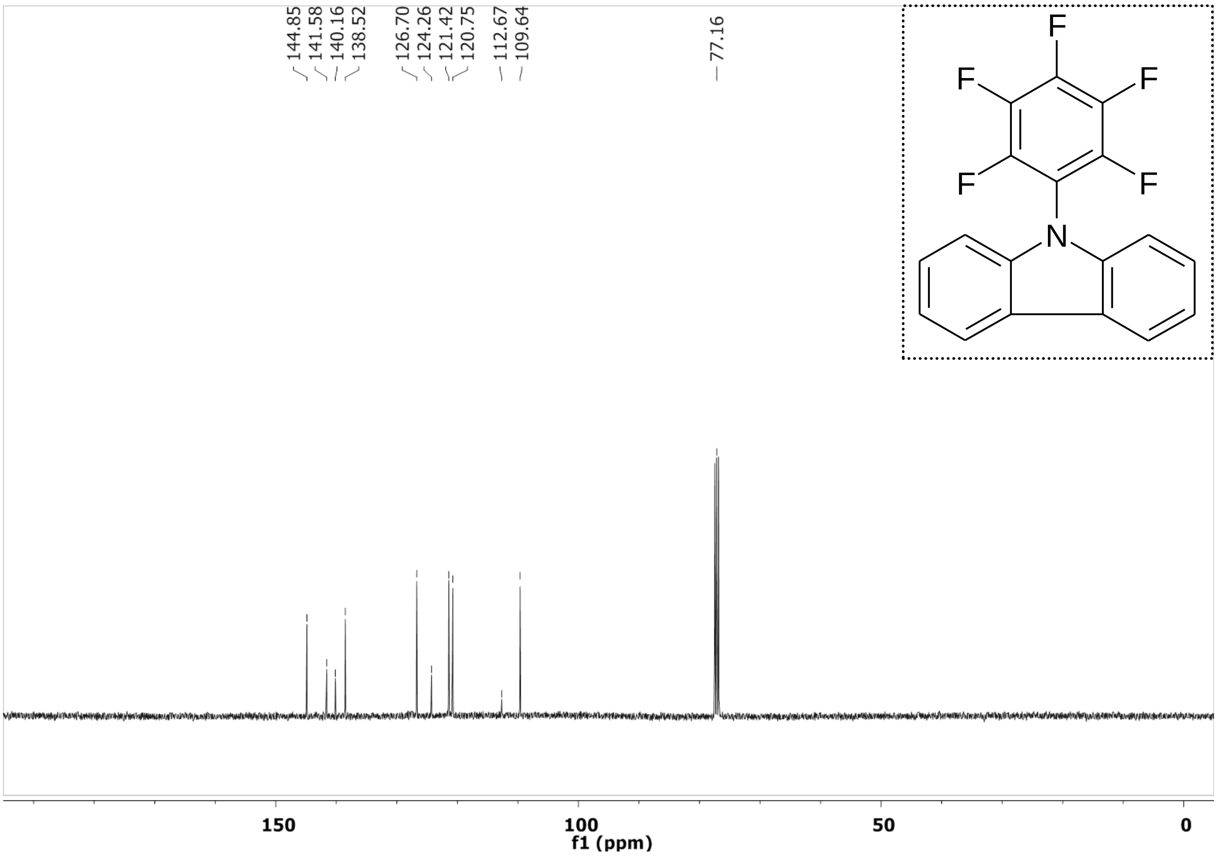

9-(Perfluorophenyl)-9*H*-carbazole (3p)

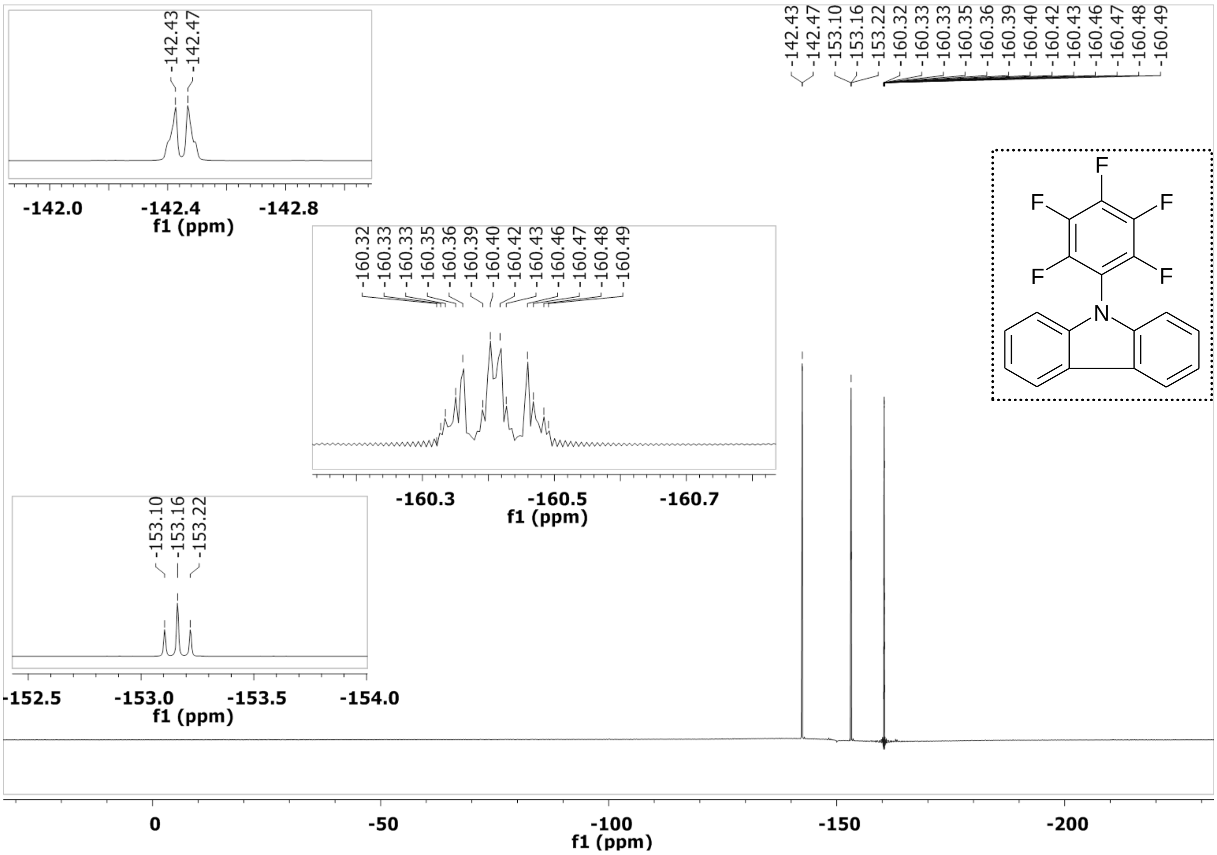

v

2’-Bromo-*N*-(4-fluorophenyl)biphenyl-2-amine (7a)

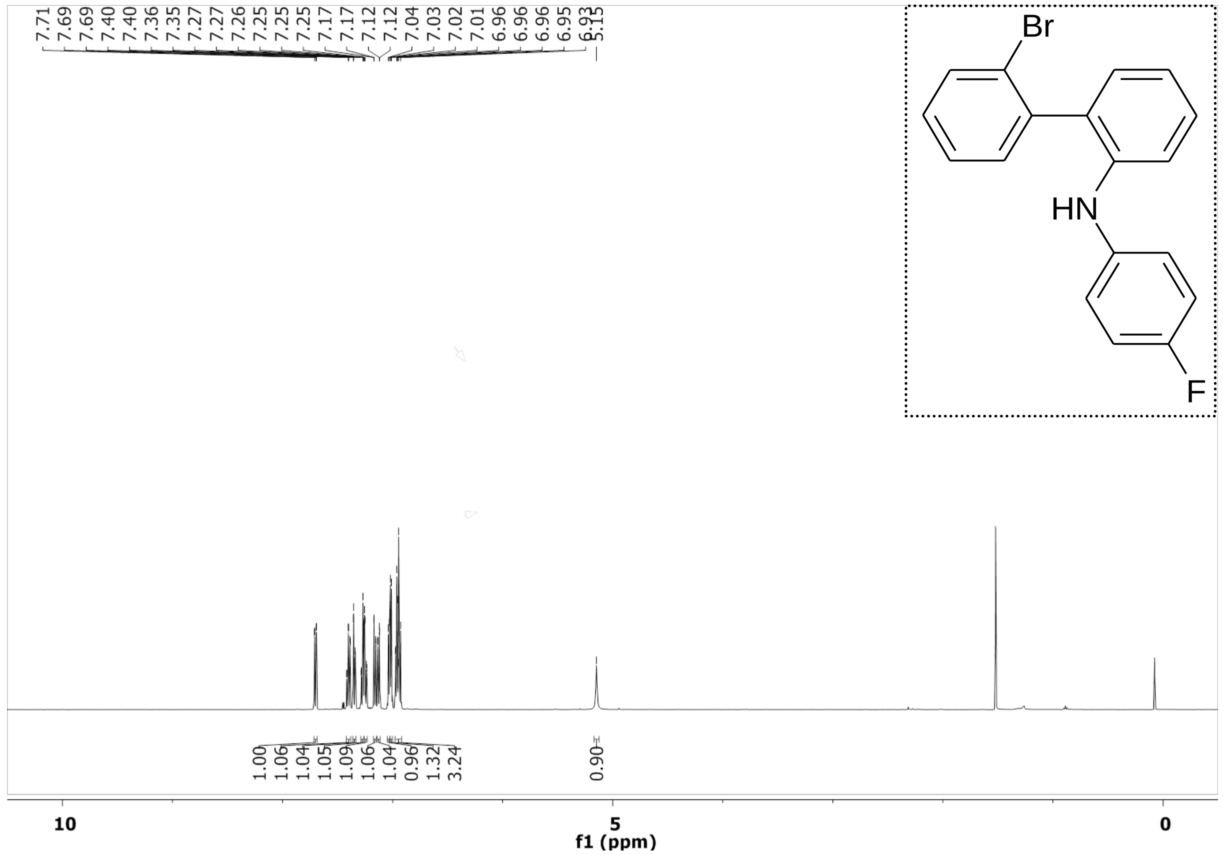

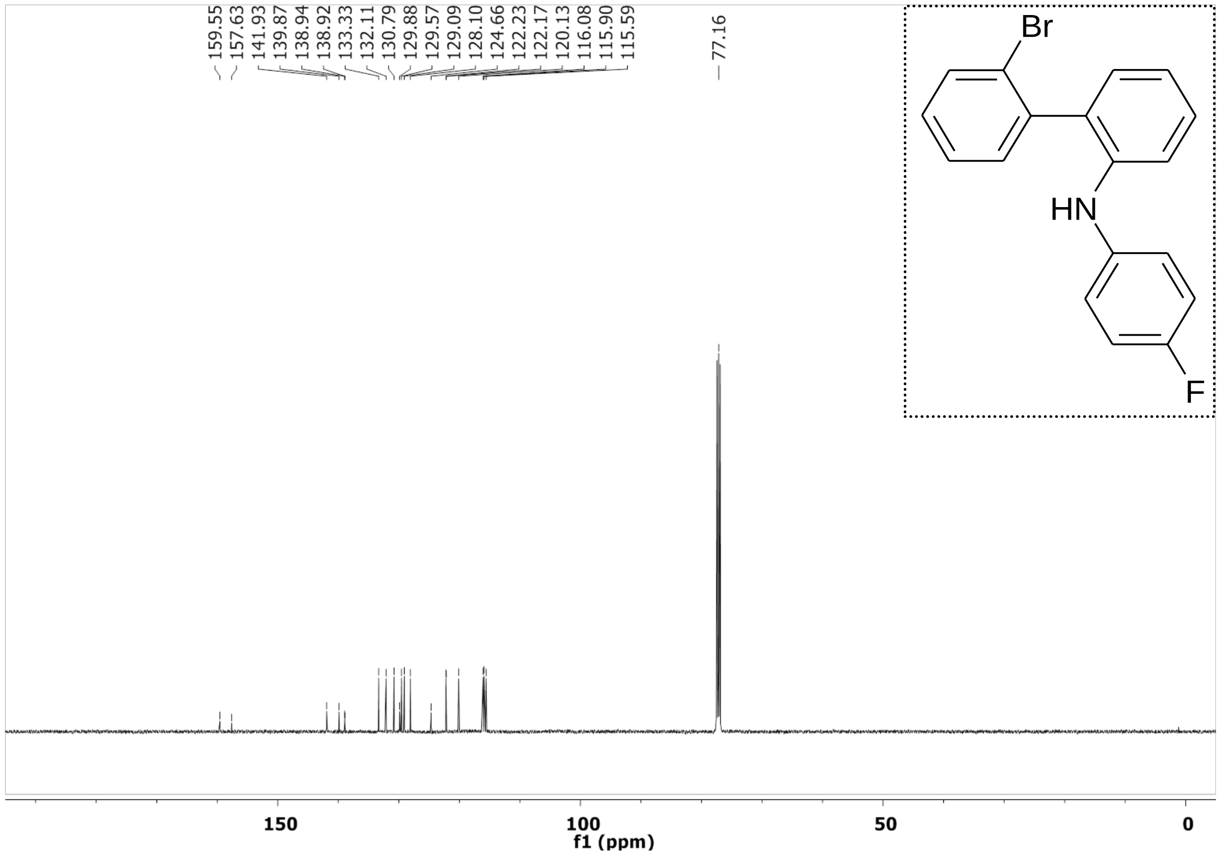

2’-Bromo-*N*-(4-fluorophenyl)biphenyl-3-amine (7b)

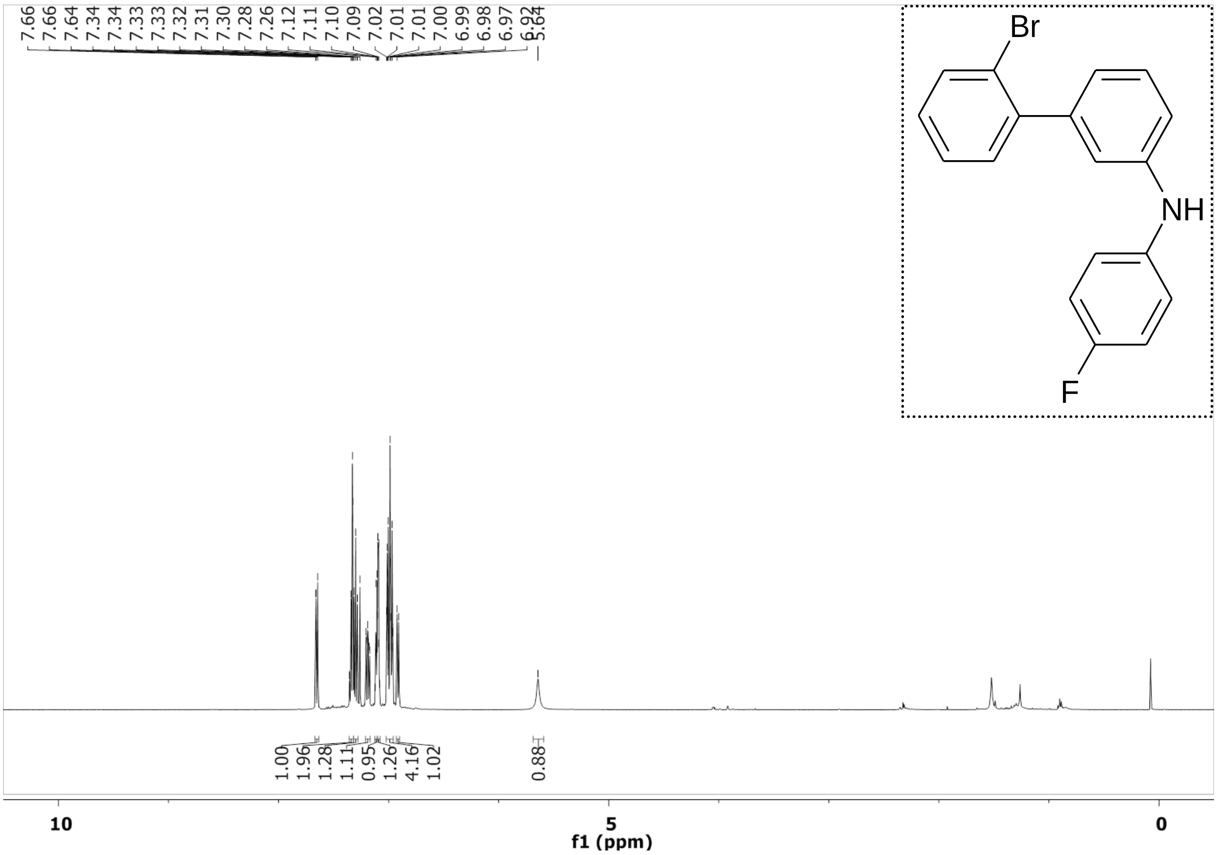

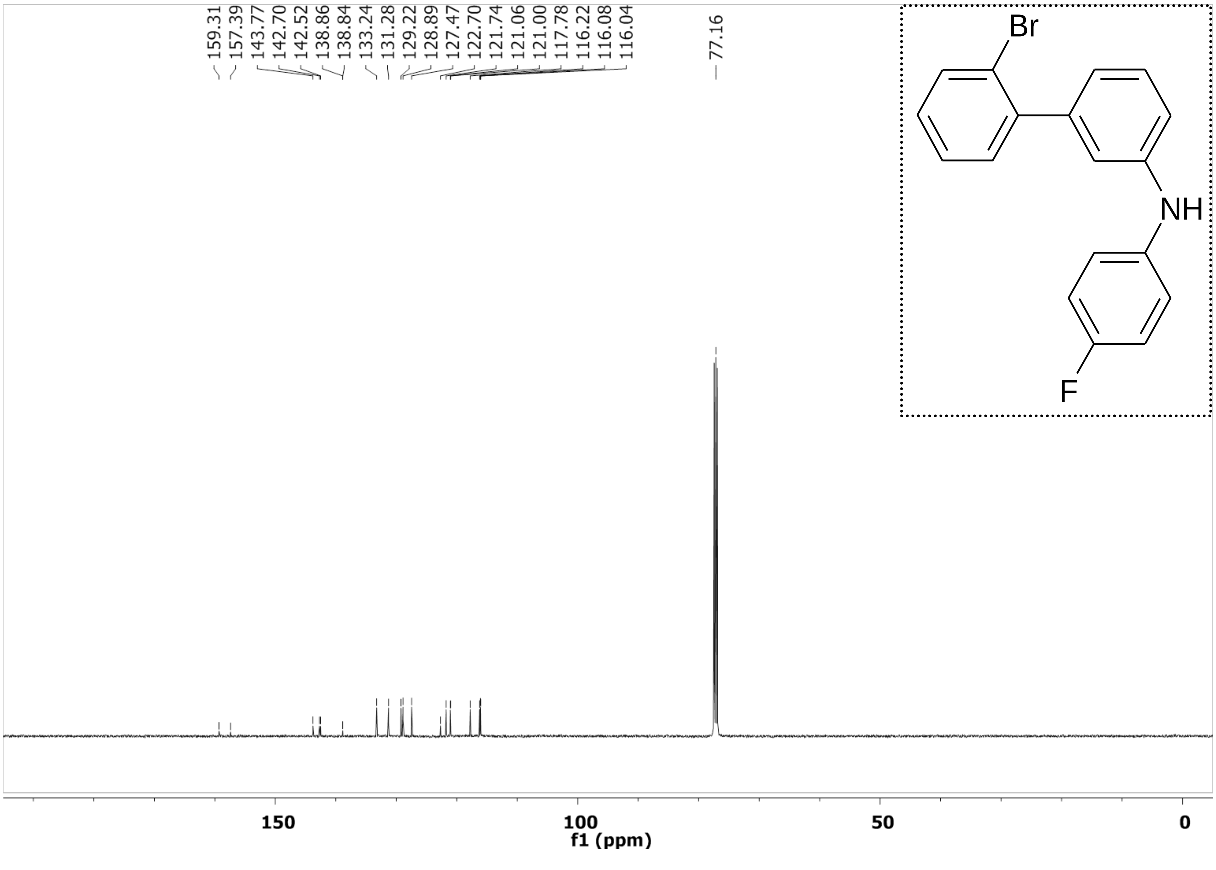

Supplement: File 1 — Experimental procedures and data of characterization of the described compounds. [file Beilstein_J_Org_Chem-09-1202-s001.pdf]
